# Tonic and burst-like locus coeruleus stimulation distinctly shift network activity across the cortical hierarchy

**DOI:** 10.1038/s41593-024-01755-8

**Published:** 2024-09-16

**Authors:** Christina Grimm, Sian N. Duss, Mattia Privitera, Brandon R. Munn, Nikolaos Karalis, Stefan Frässle, Maria Wilhelm, Tommaso Patriarchi, Daniel Razansky, Nicole Wenderoth, James M. Shine, Johannes Bohacek, Valerio Zerbi

**Affiliations:** 1 Neural Control of Movement Lab, Department of Health Sciences and Technology, ETH Zürich, Zürich, Switzerland; 2 Neuro-X institute, School of Engineering (STI), EPFL, Lausanne, Switzerland; 3 CIBM Center for Biomedical Imaging, Lausanne, Switzerland; 4 Laboratory of Molecular and Behavioral Neuroscience, Institute for Neuroscience, Department of Health Sciences and Technology, ETH Zürich, Zürich, Switzerland; 5 Neuroscience Center Zürich, ETH Zürich and University of Zürich, Zürich, Switzerland; 6 School of Physics, The University of Sydney, Sydney, New South Wales Australia; 7 Brain and Mind Centre, The University of Sydney, Sydney, New South Wales Australia; 8 Friedrich Miescher Institute for Biomedical Research, Basel, Switzerland; 9 Sorbonne Université, Institut du Cerveau-Paris Brain Institute-ICM, Inserm, CNRS, APHP, Hôpital de la Pitié Salpêtrière, Paris, France; 10 Translational Neuromodeling Unit (TNU), Institute for Biomedical Engineering, University of Zürich & ETH Zürich, Zürich, Switzerland; 11 Chemical Neuropharmacology, Institute of Pharmacology and Toxicology, University of Zürich, Zürich, Switzerland; 12 Institute for Biomedical Engineering, Department of Information Technology and Electrical Engineering, ETH Zürich, Zürich, Switzerland; 13 Institute of Biological and Medical Imaging (IBMI), Technical University of Munich and Helmholtz Center Munich, Munich, Germany; 14 Department of Psychiatry, Faculty of Medicine, University of Geneva, Geneva, Switzerland; 15 Department of Basic Neurosciences, Faculty of Medicine, University of Geneva, Geneva, Switzerland

**Keywords:** Neural circuits, Stress and resilience

## Abstract

Noradrenaline (NA) release from the locus coeruleus (LC) changes activity and connectivity in neuronal networks across the brain, modulating multiple behavioral states. NA release is mediated by both tonic and burst-like LC activity. However, it is unknown whether the functional changes in target areas depend on these firing patterns. Using optogenetics, photometry, electrophysiology and functional magnetic resonance imaging in mice, we show that tonic and burst-like LC firing patterns elicit brain responses that hinge on their distinct NA release dynamics. During moderate tonic LC activation, NA release engages regions associated with associative processing, while burst-like stimulation biases the brain toward sensory processing. These activation patterns locally couple with increased astrocytic and inhibitory activity and change the brain’s topological configuration in line with the hierarchical organization of the cerebral cortex. Together, these findings reveal how the LC–NA system achieves a nuanced regulation of global circuit operations.

## Abstract (web summary)

Tonic and burst-like locus coeruleus firing distinctly tune brain topology toward associative and sensory regions, recruiting both astrocytic and neuronal inhibitory activity.

## Main

The brain’s capacity to rapidly shift between different modes of information processing while working with limited resources supports an array of behavioral goals and contextual demands in a dynamic environment. Thus, the brain is required to orchestrate changes in the state of its neural network to manage the efficiency of specific circuit interactions. To make the process deterministic and not random, the brain must spend a certain amount of energy to engage in a state transition, the level of which can be modulated internally, much like in a catalyzed system^1^. Such a prerequisite raises a fundamental question: which neural structures and mechanisms can modulate the probability of state shifts, while stably balancing ongoing behavior and rapid adaptation? Over recent years, several theories pointed to the locus coeruleus–noradrenaline (LC–NA) system and its neuromodulatory actions as a potential candidate^2,3^. Located in the pontine brain stem with wide projections throughout the whole brain, the LC is ideally situated to accommodate the critical demands of an ever-changing environment^4,5^. It is the principal site of synthesis of the neuromodulator NA and is recognized to contribute to many central nervous system functions, including sensory processing, modulation of arousal, nociception, sleep and wake cycles, cognition and stress responses^6^.

A commonly held view is that the inherent patterns of LC activity are key drivers of several behavioral processes that can operate on subsecond timescales (for example, orienting attention) up to minutes or hours (for example, vigilance, stress)^7^. Although mostly referred to as distinct modes of firing, tonic and phasic firing probably present extremes of a continuum of LC function^2^. In monkeys, rats and mice, spontaneous LC discharges across a range of relatively low frequency (0.5–8 Hz, tonic activity) can be coarsely related to levels of arousal and focus^5,8–11^. In contrast, brief bursts of activity (2–4 spikes at 10–25 Hz) occur in association with salient or new stimuli^10–13^ and are thought to facilitate behavioral responses linked to environmental exploration and sensory acuity by increasing the neural gain in target regions at task-relevant time points^2,14^. Analyses and modeling of functional magnetic resonance imaging (fMRI) signals during pharmacological challenges, behavioral tasks or even at rest^15,16^ are in agreement with this view and have provided further evidence that LC activity levels influence the dynamics of brain states^17^. For example, periods of focused and sustained attention, compatible with moderate tonic firing of the LC, rapidly recruit the prefrontal and posterior parietal cortex and result in an improvement of executive function in humans^15^. Instead, exposing individuals to strong stressors, which are associated with high-tonic LC activity, causes rapid disengagement of prefrontal areas and induces the mobilization of the amygdala (AMY) and sensorimotor cortices to promote hypervigilance and threat detection, albeit at the expense of executive control^18,19^. More recent analyses from high-resolution, 7T resting-state human fMRI datasets further established a correlation between LC activity and the likelihood of a network’s state transition^20,21^.

The involvement of NA signaling in the control of the dynamics of connectivity states was confirmed using pharmacological blockade of adrenergic receptors^22^. However, the causal contribution of the LC to shaping brain dynamics has been investigated only recently, as chemogenetic modulation of the LC in mice was sufficient to trigger a rapid remodeling of network connectivity^23,24^. This work also revealed that adrenergic receptor distribution probably explains some of the regional specificity that noradrenergic neuromodulation can achieve, despite the diffuse projection pattern of the LC^23^. However, because of the technical constraints of chemogenetics, these studies were not able to address the key question of whether, and how, different activity patterns of the LC can shape brain network dynamics.

In this work, we set out to gain a fundamental understanding of the neural bases of LC-guided mechanisms in brain activity changes and dynamic network reconfigurations. We used optogenetics to mimic physiologically relevant excitatory patterns of tonic and burst-like LC firing while performing pupillometry, measuring local NA release with fiber photometry or recording whole-brain fMRI responses.

Our data revealed that different LC stimulation paradigms induce measurably distinct levels of downstream NA release. Contingent on these NA levels, we observed changes in widespread activity. Furthermore, we provide empirical evidence that intensity-matched tonic and burst-like LC stimulations dynamically shift network processing in the forebrain by segregating cortical regions of lower and higher hierarchical order. Taken together, these results suggest a causal involvement of the LC–NA system in shaping cortical and subcortical dynamics through its distinct firing and NA release patterns.

## Results

### LC triggers pupil response and NA release by firing pattern

**Fig. 1:**
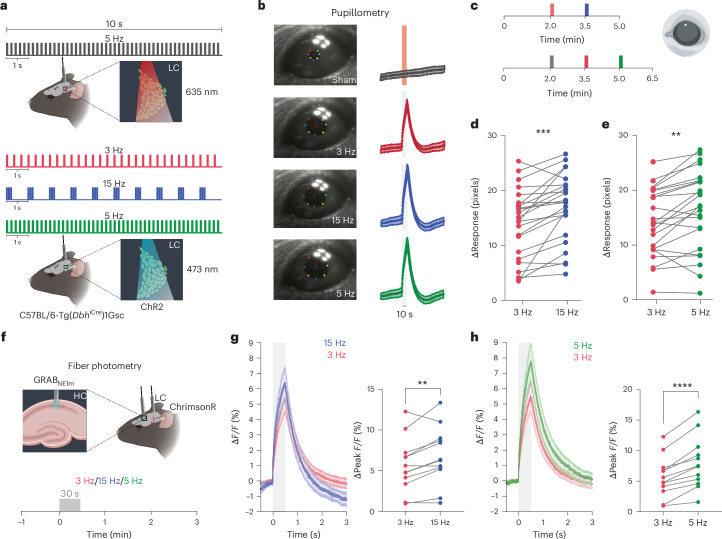
Physiological effects of LC firing intensity and pattern. **a**, Schematic of 3-Hz tonic, 15-Hz burst-like and 5-Hz tonic LC blue and red laser light (sham) stimulation protocols for pupillometry in ChR2-expressing animals. The protocol included a 2-min baseline recording followed by 10-s stimulations: (1) 5 Hz at 635 nm as control; (2) 3 Hz at 473 nm; (3) 15 Hz at 473 nm; and (4) 5 Hz at 473 nm. **b**, Left, representative images during 10-s sham, 3-Hz, 15-Hz and 5-Hz LC stimulation. Right, corresponding pupil traces (*n* = 24, mean ± s.e.m.). **c**, Schematic of the optogenetic LC stimulation protocol for pupillometry in ChR2-expressing animals (color-coded according to **a**). **d**, Statistical comparison of the pupil response to tonic and burst-like LC stimulation revealed that at 15 Hz changes in pupil diameter were significantly greater than at 3 Hz (mean ± s.d. for 3 Hz = 13.89 ± 6.37; mean ± s.d. for 15 Hz = 17.02 ± 6.13; two-sided paired *t*-test, *t*_(23)_ = 4.56, *P* = 0.00014; *n* male mice = 12, *n* female mice = 12). **e**, Statistical comparison of the pupil response to 3-Hz and 5-Hz tonic LC stimulation. A 5-Hz LC stimulation induced a significantly greater pupil dilation than 3 Hz (mean ± s.d. for 3 Hz = 14.24 ± 6.1; mean ± s.d. for 5 Hz = 16.47 ± 7.3; two-sided paired *t*-test, *t*_(23_) = 3.7, *P* = 0.0012; *n* male mice = 12, *n* female mice = 12). **f**, Schematic of 30-s optogenetic LC stimulation (ChrimsonR) combined with fiber photometry in the HC. **g**, Left, Δ*F/F* traces of GRAB_NE1m_ photometry recordings in response to tonic 3-Hz and 15-Hz burst-like LC stimulation (mean ± s.e.m.). Right, 15-Hz burst-like LC stimulation triggered greater NA release compared to 3-Hz tonic LC stimulation (mean ± s.d. for 3 Hz = 5.64 ± 3.30; mean ± s.d. for 15 Hz = 6.82 ± 3.54; two-sided paired *t*-test, *t*_(11)_ = 3.42, *P* = 0.0058; *n* male mice = 3, *n* female mice = 9). **h**, Left, Δ*F/F* traces of GRAB_NE1m_ photometry recordings in response to tonic 3-Hz and 5-Hz LC stimulation (mean ± s.e.m.). Right, 5-Hz tonic LC stimulation triggered greater NA release compared to 3-Hz tonic LC stimulation (mean ± s.d. for 3 Hz = 5.64 ± 3.30; mean ± s.d. for 5 Hz = 7.97 ± 4.22; two-sided paired *t*-test, *t*_(11)_ = 6.53, *P* = 0.000043; *n* male mice = 3, *n* female mice = 9). **P < 0.01, ***P < 0.001, ****P < 0.0001. Figure schematics in panels **a**, **c**, and **f** were created with BioRender.com.

**Extended Data Fig. 1:**
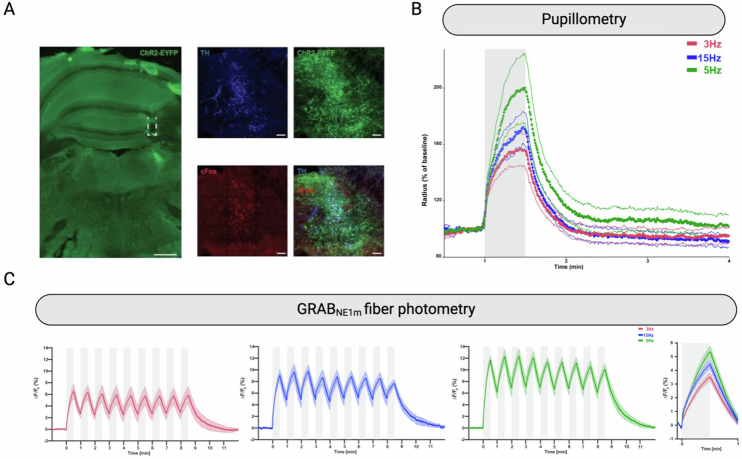
Physiological effects of 30 s optogenetic LC-NA stimulation. Related to Fig. 1. **a** Left: immunohistochemical macro view of ChR2-EYFP targeting to the right (ipsilateral) LC. Dashes represent optical fibre placement. Right: immunohistochemical view of ChR2-EYFP and cFos expression in TH-positive neurons of the right (stimulated) LC. Immunohistochemistry was performed in a separate cohort to evaluate LC targeting (n(male)=11). **b** Pupil traces at 30 s of 3 Hz tonic (n=9), 15 Hz burst (n=9), and 5 Hz tonic (n=7) LC stimulation (mean±SEM). **c** GRAB_NE1m_ photometry recordings during nine blocks of 30 s 3 Hz tonic, 15 Hz burst, and 5 Hz tonic LC ChrimsonR stimulation. Each stimulation block causes a sharp spike in NA increase across all stimulation protocols. Right: GRAB_NE1m_ photometry recordings during 30 s 3 Hz tonic, 15 Hz burst, and 5 Hz tonic LC ChrimsonR stimulation averaged across nine stimulation blocks (mean±SEM). Scale bars, 500µm,50µm. N(male)=2, n(female)=3. Figure schematics in panels **b** and **c** were created with Biorender.com.

To achieve spatially and temporally precise modulation of LC noradrenergic neurons, we unilaterally transfected the right LC of *Dbh*^iCre^ mice (*n* male mice = 22; *n* female mice = 7) with an adeno-associated virus (AAV) construct carrying an optogenetic actuator and implanted an optical cannula above the LC (Extended Data Fig. 1a). We first assessed the physiological impact of optogenetically evoked tonic and burst-like LC firing using pupillometry^23^. A 2-min baseline recording was followed by different laser stimulations as shown in Fig. 1a,c. We chose a 3-Hz tonic frequency because several studies suggested that this intermediate level of LC activity is associated with optimal task performance^2,25–27^. To mimic the natural burst activity reported in LC neurons^11–13^, we chose a subsecond 15-Hz stimulation frequency, where the total number of stimulation pulses delivered was matched to the tonic 3-Hz stimulation (Fig. 1a). In this ‘intensity-matched’ design, 10-s burst-like LC stimulation at 15 Hz evoked pupil dilations that were larger than at 3-Hz tonic LC stimulation (mean ± s.d. for 3 Hz = 13.89 ± 6.37, mean ± s.d. for 15 Hz = 17.02 ± 6.13; paired *t*-test, *t*_(23)_ = 4.56, *P* < 0.0001) (Fig. 1d), suggesting that despite intensity-matched stimulation conditions, tonic and burst-like LC firing patterns differentially affect physiological responses. No pupil dilation occurred during red-light sham stimulation (Fig. 1b).

Next, we tested how different frequencies (intensities) of tonic LC firing affect pupillary responses. To this end, we chose a 5-Hz tonic frequency (Fig. 1a) as earlier research demonstrated that tonic 5-Hz stimulation closely mimics the effects of intense acute stressors^28^ while higher stimulation frequencies (for example, 10 Hz) may actually result in NA depletion or LC exhaustion^17^. When activating the LC with 5-Hz tonic stimulation, changes in pupil size were greater than at 3 Hz (mean ± s.d. for 3 Hz = 14.24 ± 6.1; mean ± s.d. for 5 Hz = 16.47 ± 7.3; paired *t*-test, *t*_(23)_ = 3.7, *P* = 0.0012) (Fig. 1e), suggesting that increasing the intensities of tonic LC–NA activity trigger a graded pupillary response. After all three LC stimulation protocols, baseline levels of pupil diameter were re-established within a time frame of 30 s (Fig. 1b and Extended Data Fig. 1b).

To assess whether different stimulation patterns would lead to different levels of NA release, we performed fiber photometry recordings in the hippocampus (HC), a brain region receiving noradrenergic input exclusively from the LC^29^ (Fig. 1f). In the intensity-matched condition (3 Hz versus 15 Hz), 30 s of a 15-Hz burst stimulation elicited greater NA release compared to tonic 3-Hz stimulation (mean ± s.d. for 3 Hz = 5.64 ± 3.30; mean ± s.d. for 15 Hz = 6.82 ± 3.54; paired *t*-test, *t*_(11)_ = 3.415, *P* = 0.0058; Fig. 1g). In keeping with earlier findings that showed linear scaling of NA release with tonic LC activity up to 6 Hz^30,31^, the change in fluorescence of the NA sensor GRAB_NE1m_^32^ was greater in response to the higher-intensity 5-Hz tonic stimulation compared to 3-Hz tonic LC stimulation (mean ± s.d. for 3 Hz = 5.64 ± 3.30; mean ± s.d. for 5 Hz = 7.97 ± 4.22; paired *t*-test, *t*_(11)_ = 6.528, *P* < 0.0001; Fig. 1h).

To devise a stimulation paradigm that would allow us to robustly assess brain-wide activity levels using block-design fMRI, we then introduced nine blocks of 30-s laser stimulation, with each block interleaved by a 30-s rest period. Using this design, we additionally monitored NA release to determine whether NA depletion or accumulation would occur over the course of recurrent, prolonged optogenetic stimulation. Each block caused a sharp increase in GRAB_NE1m_ fluorescence that peaked at the end of the stimulation block, suggesting that NA release could be reliably triggered over time and that no NA buildup occurred after repeated optogenetic stimulation (Extended Data Fig. 1c). Although NA traces did not fully recover during the laser OFF blocks, this effect is probably attributable to the slow off-kinetics of the GRAB_NE1m_ sensor rather than insufficient NA clearance, as suggested by the full recovery of pupil diameter (Fig. 1b and Extended Data Fig. 1b).

### Tonic and burst-like LC stimulation evokes biphasic blood oxygen level-dependent responses

**Fig. 2:**
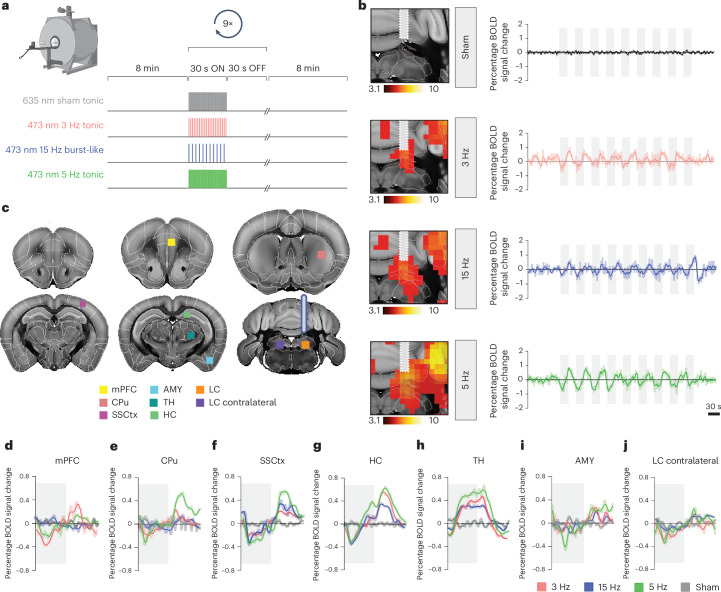
Local effects of optogenetic LC–NA stimulation. **a**, Schematic of tonic and burst-like blue-light, red-light and sham laser stimulation protocols for combined LC optogenetic-fMRI. **b**, Mean time series extracted from the targeted right LC from sham, 3-Hz tonic, 15-Hz burst-like and 5-Hz tonic stimulation groups (mean ± s.e.m.). **c**, ROI location for mean time series extraction. LC projections were targeted toward most forebrain regions^61^; hence, no other a priori hypothesis as to the anatomical location of the portrayed ROIs was made. **d**–**j**, Percentage of changes in BOLD signal of the time series extracted from the medial prefrontal cortex (mPFC) (**d**), caudate putamen (CPu) (**e**), SSCtx (**f**), HC (**g**), thalamus (TH) (**h**), AMY (**i**) and contralateral LC (**j**) averaged across nine stimulation blocks. The gray-shaded areas represent laser stimulation ON blocks. *n* sham = 32; *n* 3 Hz = 15; *n* 5 Hz = 16; *n* 15 Hz = 18. Figure schematics in panels **a**–**c** were created with BioRender.com.

**Extended Data Fig. 2:**
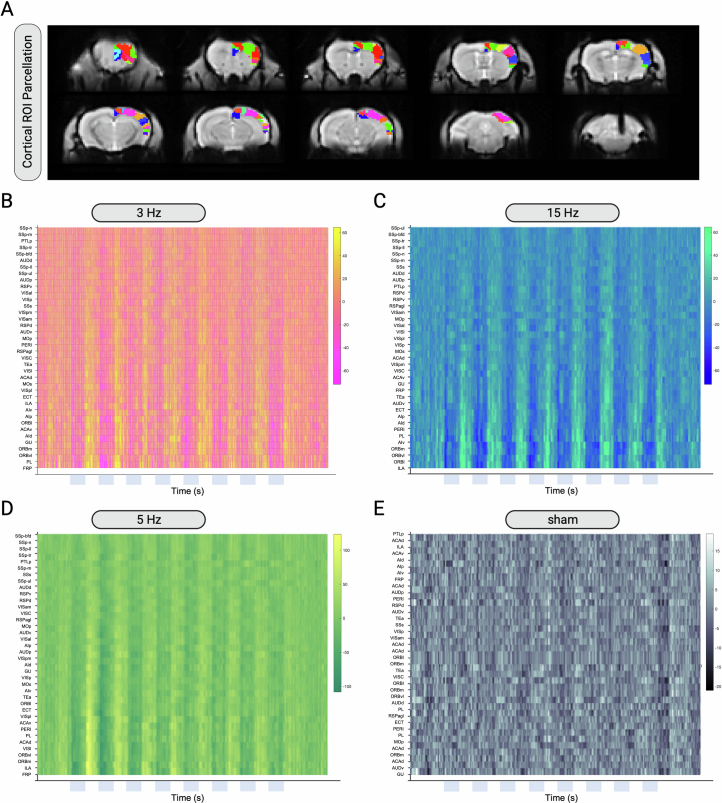
Cortical ROI parcellation. Related to Fig. 2. **a** Location of 38 regions-of-interest (ROIs) in EPI template space across the right cortical hemisphere used for time series extraction. Mean timeseries of 38 cortical ROIs during **b** 3 Hz tonic, **c** 15 Hz burst, **d** 5 Hz tonic, and **e** sham LC stimulation. Figure schematics in panels **a**–**c** were created with Biorender.com.

Next, we assessed the whole-brain, time-locked effects of tonic and burst-like LC firing using a combined optogenetic-fMRI approach with blue-light stimulation at an intensity-matched 3-Hz tonic and 15-Hz burst as well as 5-Hz tonic frequency, and in response to a red-light control stimulation (Fig. 2a). Because of the neuromodulatory and vasoconstrictive effects of NA^24^, we suspected that our optogenetic LC stimulation may decouple the typical functional hyperemic response captured by blood oxygen level-dependent (BOLD) fMRI. To test this, we first extracted the mean time series from the targeted right LC and found that each block of optogenetic laser pulses repeatedly triggered a biphasic response, with an initial drop in BOLD signals followed by a progressive increase, which reverted to baseline levels during the laser offset time (maximum percentage range; Fig. 2b). This biphasic response was consistent across all three stimulation conditions and across most regions of interest (ROIs) sampled along the anterior-posterior brain axis and ipsilateral to the stimulated site (Fig. 2c–i and Extended Data Fig. 2a–d). No changes in signal were detected during the red-light sham stimulations (maximum percentage range; Fig. 2b–j and Extended Data Fig. 2e). Qualitative inspection of the time series revealed that the BOLD signal changes (calculated as the percentage change from baseline) varied among the sampled ROIs depending on the LC stimulation patterns and intensities (Fig. 2d–j, averaged across nine stimulation blocks). These results suggest that different LC firing intensities and patterns affect activity in target brain regions selectively and nonlinearly.

### LC–NA modulates biphasic BOLD via inhibition and astrocytes

**Fig. 3:**
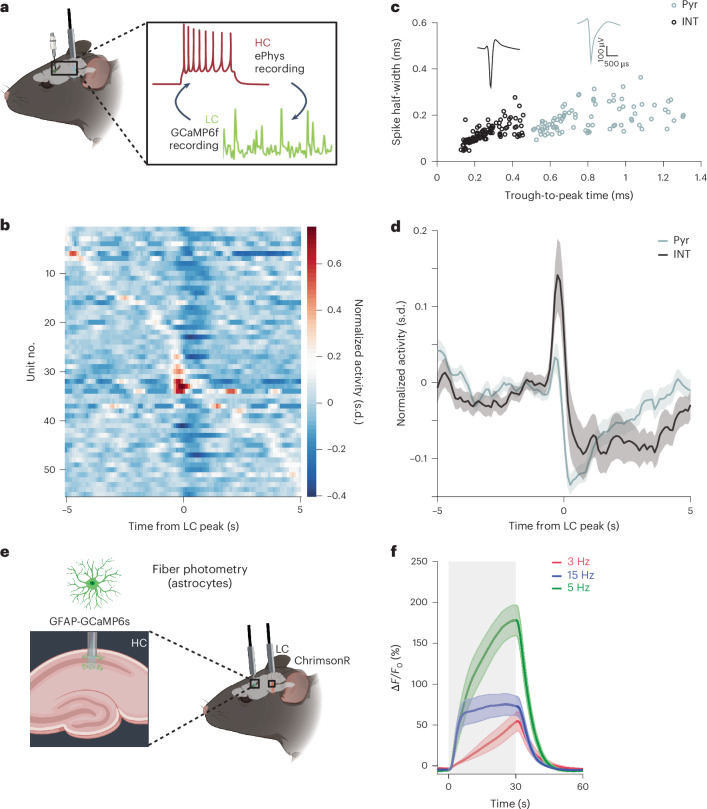
Effects of LC–NA activity on neuronal and astrocytic activity. **a**, Electrophysiological recordings of the HC in awake, head-restrained mice. Simultaneously, spontaneous Ca^2+^ transients were recorded in the right LC using GCaMP6f. **b**, Color-coded raster plots of the activity of hippocampal neurons after intrinsic GCaMP6f activation of the LC. **c**, Clustering of extracellularly recorded units into putative principal and fast-spiking INTs, and representative spike waveforms. **d**, Mean firing rate of the principal neuron population (gray) and INT population (black) in the HC represented as ± s.e.m. **e**, Schematic of 30-s optogenetic LC stimulation (ChrimsonR) combined with GFAP-GCaMP6s fiber photometry recording in the HC. **f**, Δ*F/F* traces of astrocytic Ca^2+^ traces averaged across nine 30-s stimulation blocks of 3-Hz tonic, 15-Hz burst-like and 5-Hz tonic LC stimulation represented as ± s.e.m. *n* Ca^2+^ = 4; *n* ePhys = 3. Pyr, pyramidal neuron. Figure schematics in panels **a** and **e** were created with BioRender.com.

To further explore the biphasic nature of the observed BOLD response, we looked at the impact of LC–NA activity on key components of the neurovascular unit, namely neurons and astrocytes. To this end, we first measured transients in LC activity using the calcium indicator GCaMP6f while simultaneously recording electrophysiological activity in the HC of awake, head-fixed mice (Fig. 3a). Through the temporal synchronization of LC GCaMP6f peaks with the electrophysiological response of hippocampal neurons, we discovered that during these transients, subsets of the recorded neurons exhibited inhibitory responses, whereas a small subset of neurons showed increased excitation (Fig. 3b). We classified the recorded neurons into putative excitatory principal neurons and fast-spiking inhibitory interneurons (INTs) based on their extracellular waveforms^33^ (Fig. 3c). This revealed that primarily INTs were activated, whereas excitatory cells were inhibited in response to LC transients (Fig. 3d), alluding to a potential mechanism underlying the prominent BOLD signal drop on laser stimulation onset^34–36^.

**Extended Data Fig. 3:**
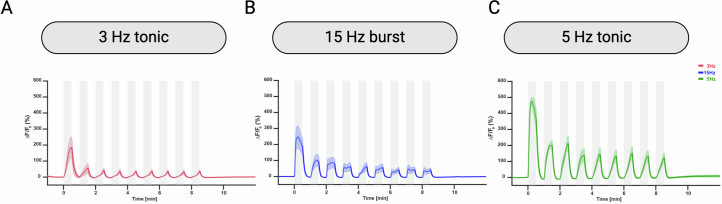
Astrocytic activity upon repeated optogenetic LC-NA stimulation. Related to Fig. 3. Changes in astrocytic Ca^2+^ release upon nine blocks of 30 s optogenetic **a** 3 Hz tonic, **b** 15 Hz burst, and **c** 5 Hz tonic LC ChrimsonR stimulation recorded in the hippocampus (mean±SEM). Each stimulation block causes an increase in Ca^2+^ signals across all stimulation protocols. N(male)=2, n(female)=2. Pink traces represent 3 Hz tonic, green traces 5 Hz tonic, blue traces 15 Hz burst, and grey blocks indicate optogenetic stimulation. Figure schematics in panels **a**–**c** were created with Biorender.com.

We then measured LC stimulation-evoked astrocytic activity (Fig. 3e), a prime target of NA signaling^37^. Glial fibrillary acidic protein (GFAP)-GCaMP6s recordings conducted in hippocampal astrocytes demonstrated that on each of the stimulation protocols there was an elevation in Ca^2+^ signals that reached a peak on stimulation cessation (mean ± s.e.m.; Fig. 3f and Extended Data Fig. 3a–c). Notably, this increase in signal was much more enhanced compared to any neuronal response upon LC–NA activity. This observation suggests a potential association between the recruitment of a greater number of astrocytes because of sustained LC–NA activity and the observed enhancement in BOLD signals^38–40^. In line with pupil diameter and NA release dynamics, averaged Ca^2+^ signals increased from 3-Hz tonic to 15-Hz burst-like to 5-Hz tonic stimulation (Fig. 3f) and returned to baseline within 30 s.

Our collective findings demonstrate that the LC–NA system influences BOLD responses through two parallel potential mechanisms: modulation of inhibitory transmission (as supported by previous studies^41^) and gradual recruitment of astrocytes. These mechanisms have the potential to drive distinct hemodynamic responses in the observed BOLD signals.

### Tonic and burst-like LC stimulation evoke distinct BOLD activity

**Fig. 4:**
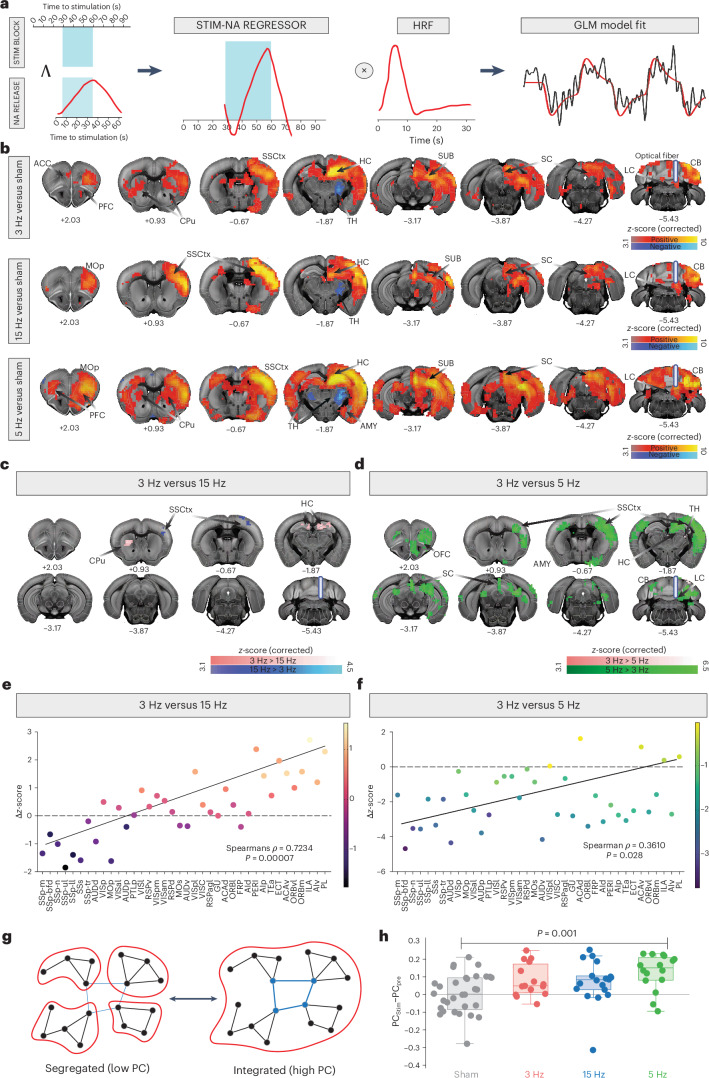
Brain-wide effect of tonic and burst-like LC stimulation. **a**, GLM model setup, including the STIM-NA agreement regressor between the LC ON/OFF laser stimulation protocol (that is, STIM BLOCK) and the NA stimulus responses (that is, NA RELEASE) as obtained from the summation of both parameter estimates (using the logical ‘AND’ function). The agreement regressor (that is, STIM-NA REGRESSOR) was convolved with a standard double-gamma HRF, resulting in an ideal model fit (red trace) of the fMRI data (black trace; taken from the strongest voxel in the targeted LC for visualization purposes). **b**, Cluster-corrected, thresholded GLM *z*-statistic activation maps of the 3-Hz tonic, 15-Hz burst and 5-Hz tonic groups compared to sham (*z*-statistic images, Gaussianized T/F) were thresholded nonparametrically using clusters determined by *z* > 3.1 and a (corrected) cluster significance threshold of *P* = 0.05. **c**, Selective activation clusters during 3-Hz tonic (pink) and 15-Hz burst-like (blue) stimulation of the LC (*z*-statistic images, Gaussianized T/F) were thresholded nonparametrically using clusters determined by *z* > 3.1 and a (corrected) cluster significance threshold of *P* = 0.05. **d**, Selective activation clusters during 3-Hz tonic (pink) and 5-Hz tonic (green) stimulation of the LC (*z*-statistic images, Gaussianized T/F) were thresholded nonparametrically using clusters determined by *z* > 3.1 and a (corrected) cluster significance threshold of *P* = 0.05. **e**, Changes in *z*-scores relative to the activation of cortical hierarchical regions during 3-Hz tonic versus 15-Hz burst-like LC activation (Spearman *ρ* = 0.7234, *P* < 0.000). **f**, Changes in *z*-scores relative to the activation of cortical hierarchical regions during 3-Hz tonic versus 5-Hz tonic LC activation (Spearman *ρ* = 0.3610, *P* = 0.028). **g**, BOLD network changes were quantified using the PC. **h**, Mean cortical PCs during LC stimulation in sham (black), 3-Hz tonic (pink), 15-Hz burst (blue) and 5-Hz tonic (green) datasets, corrected for the prestimulation baseline. Data showed significant increase in mean cortical PC from the prestimulation to the stimulation period (*P* < 0.05, Kruskal–Wallis test, corrected for multiple comparisons). A 5-Hz tonic stimulation resulted in a significantly different mean cortical PC compared to sham stimulation (*P* < 0.05, Kruskal–Wallis test, corrected for multiple comparison). The central mark indicates the median; the bottom and top edges of the box indicate the 25th and 75th percentiles, respectively. The whiskers extend to the most extreme data points not considered as outliers. The bar plots represent ± s.e.m. *n* sham = 32; *n* 3 Hz = 15; *n* 5 Hz = 16; *n* 15 Hz = 18. ACAd, anterior cingulate area, dorsal part; ACAv, anterior cingulate area, ventral part; ACC, anterior cingulate cortex; Ald, agranular insular area, dorsal part; Alp, agranular insular area, posterior part; Alv, agranular insular area, ventral part; AUDd, dorsal auditory area; AUDp, primary auditory area; AUDv, ventral auditory area; CB, cerebellum; ECT, ectorhinal area; FRP, frontal pole, cerebral cortex; GU, gustatory area; HY, hypothalamus; ILA, infralimbic area; MOp, primary motor area; MOs, secondary motor area; ORBI, orbital area, lateral part; ORBm, orbital area, medial part; ORBvl, orbital area, ventrolateral part; PERI, perirhinal area; PL, prelimbic area; PTLp, posterior parietal association area; RSPagl, retrosplenial area, lateral agranular part; RSPd, retrosplenial area, dorsal part; RSPv, retrosplenial area, ventral part; SSp-bfd, primary somatosensory area, barrel field; SSp-ll, primary somatosensory area, lower limb; SSp-m, primary somatosensory area, mouth; SSp-n, primary somatosensory area, nose; SSp-t, primary somatosensory area, trunk; SSp-ul, primary somatosensory area, upper limb; SSs, supplemental somatosensory area; TEa, temporal association area; VISal, anterolateral visual area; VISam, anteromedial visual area; VISC, visceral area; VISl, lateral visual area; VISp, primary visual area; VISpl, posterolateral visual area; VISpm, posteromedial visual area. Figure schematics in panels **a**–**g** were created with BioRender.com.

**Extended Data Fig. 4:**
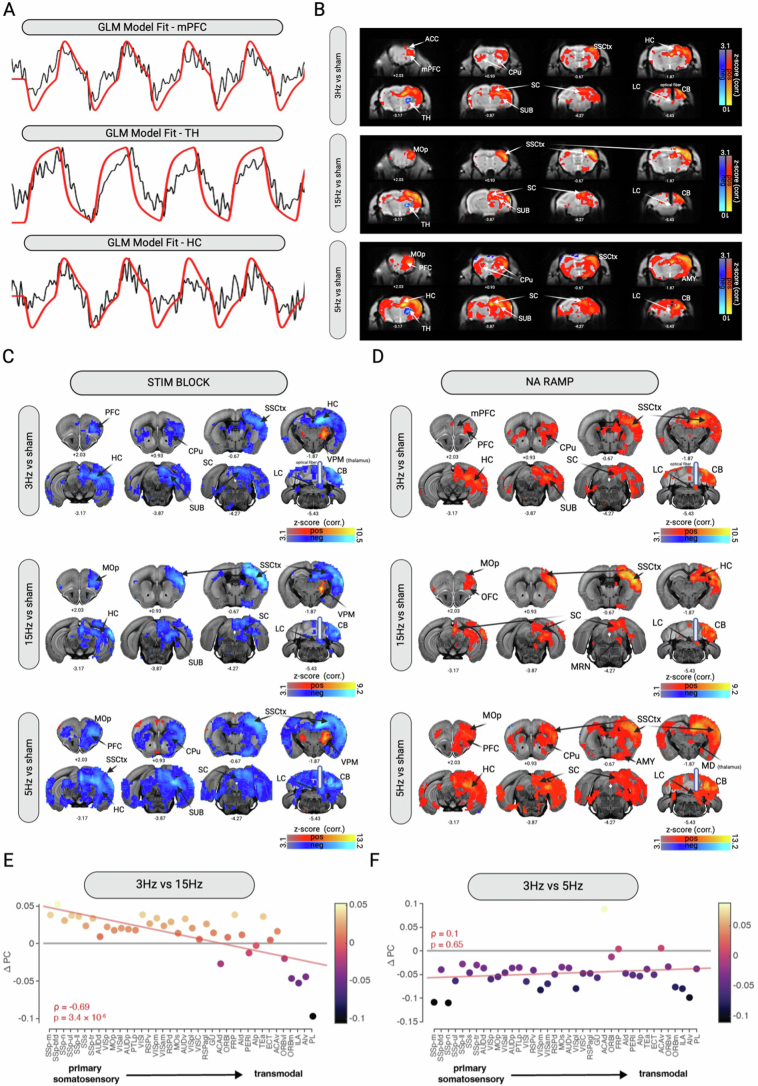
Brain-wide effects of optogenetic LC stimulation. Related to Fig. 4. **a** GLM model fit of selected ROIs. **b** Cluster-corrected, thresholded (z-score>3.1) GLM z-stat activation maps in EPI space of 3 Hz tonic, 15 Hz burst, and 5 Hz tonic groups compared to sham. General linear model (GLM) model analysis including the LC ON/OFF laser stimulation protocol (that is, STIM BLOCK) and the NA stimulus responses (that is, NA RAMP) as separate but highly correlated regressors. Both are convolved with a standard double-gamma HRF resulting in cluster-corrected, thresholded (z-score>3.1) GLM z-stat activation maps of 3 Hz tonic, 15 Hz burst, and 5 Hz tonic groups compared to sham that depend on **c** the ON OFF stimulation of LC-NA neurons or **d** on NA release dynamics. **e** Changes in participation coefficients (PC) relative to activation of cortical hierarchical regions during 3 Hz tonic vs 15 Hz burst-like LC activation (Spearman’s $\rho \,$= −0.69, *P* = 3.4${\times 10}^{-6}$). **f** Changes in participation coefficients (PC) relative to activation of cortical hierarchical regions during 3 Hz tonic vs 5 Hz tonic LC activation (Spearman’s ρ = 0.1, *P* = 0.65). PFC, prefrontal cortex; mPFC, medial prefrontal cortex; OFC, orbitofrontal cortex; CPu, caudate putamen; Amy, amygdala; HC, hippocampus; SUB, subiculum; SC, superior colliculus; LC, locus coeruleus; MRN, midbrain reticular nucleus; VPM, ventral posteriomedial nucleus of thalamus; MOp, primary motor cortex; SSCtx, somatosensory cortex; CB, cerebellum; NA, noradrenaline; Stim, stimulation. N(sham)=32; n(3Hz)=15; n(5Hz)=16. Figure schematics in panels **a**–**f** were created with Biorender.com.

After identifying two potential effects contributing to the biphasic fMRI signal, our subsequent objective was to quantify these fMRI responses upon optogenetic LC manipulations. To achieve this, a custom regressor was computed by summing two parameter estimates, that is, the block stimulation and the NA release curve derived from the average of 3-Hz tonic, 15-Hz burst-like and 5-Hz tonic NA release curves (Fig. 4a). This way, we ensured that our analysis would accurately account for the biphasic nature of our BOLD fMRI data. We combined two parameter estimates using a logical ‘AND’ function: one representing the negative hemodynamic response linked to neuronal activity induced by our block stimulation protocol; the other representing the delayed positive hemodynamic response linked to the NA release dynamics recorded with GRAB_NE1m_ (Fig. 4a). Both regressors were combined in a general linear model (GLM) and convolved with a standard double-gamma hemodynamic response function (HRF). We found that this approach achieved an optimal model fit across most brain regions activated during our three LC stimulation protocols (Fig. 4a and Extended Data Fig. 4a; *z* > 3.1, cluster-corrected). Next, we used our GLM framework to generate group activation maps of each stimulation type and compared them to sham controls. This revealed that most ipsilateral and contralateral brain regions were aligned to our model, while signals in thalamic nuclei showed a negative correlation in each of the three LC–NA stimulation patterns (Fig. 4b and Extended Data Fig. 4b). On qualitative examination of each regressor’s influence independently, we observed that the BOLD signal in the TH increased in conjunction with the onset of laser stimulation rather than NA release (Extended Data Fig. 4c,d). This suggests that LC activity promptly and continuously enhances thalamic excitability^31^, evoking a positive hyperemic response that is consistent with previous electrophysiological findings^42^. Conversely, all other brain regions exhibited contributions from both regressors, indicating a multifaceted response to LC activity.

We then directly compared the two intensity-matched conditions and saw distinct patterns of activation: while 3-Hz tonic stimulation engaged hippocampal regions, 15-Hz burst-like stimulation recruited sensory areas (Fig. 4c; *z* > 3.1, cluster-corrected). Furthermore, comparison of the two tonic stimulation intensities (3 Hz versus 5 Hz) revealed an overall increase in local BOLD responses during 5-Hz stimulation, including frontal regions, large portions of the somatosensory cortex (SSCtx) and the AMY (Fig. 4d; *z* > 3.1, cluster-corrected). To better understand which regions were primarily influenced by our specific LC firing patterns, we mapped the group contrast results onto the hierarchical functional organization of cortical areas in mice. This organization represents a gradient from lower-level sensory processing to higher regions of more abstract information (transmodal regions)^43^. We found that moderate tonic versus burst-like LC activation closely corresponded to the hierarchical cortical organization, with transmodal versus somatosensory areas recruited, respectively (Fig. 4e; Spearman $\rho$ = 0.7234). Using a two-tailed test, we statistically analyzed this correlation against a distribution of 10,000 Spearman *ρ* values generated by randomly shuffling the labels of cortical regions. Our results demonstrate that the observed linear relationship within the cortical hierarchy significantly surpassed random label assignment (*P* < 0.000). A similar, albeit weaker, gradient alignment was observed when comparing moderate tonic versus high-tonic LC activation patterns (Fig. 4f; Spearman *ρ* = 0.3610, *P* = 0.028). These findings suggest that variations in LC firing intensity and pattern exert a distinct causal influence on cortical processing, corresponding to their hierarchical ranking.

Next, we posited that the spatial patterns of brain activity changes may correlate with the expression of adrenergic receptors in the brain^23^. To test this hypothesis, we performed a correlation analysis between the results of our GLM model and the adrenergic receptor density maps obtained from the Allen Brain Institute Gene Expression Database^44^. The analysis revealed a robust spatial relationship with the adrenergic α1A receptor (Adra1a subtype) (Spearman *ρ* for 3 Hz, 15 Hz and 5 Hz = 0.33, 0.30 and 0.34; *P* < 0.0001, Bonferroni-corrected) across 3-Hz tonic, 15-Hz burst-like and 5-Hz tonic groups (Supplementary Table 1). This finding suggests that the observed BOLD effects are primarily mediated by α1A receptors. Moreover, we found a significant correlation between 5-Hz tonic LC stimulation and the adrenergic-β (Adrb) receptor (Spearman *ρ*= 0.38; *P* < 0.0001, Bonferroni-corrected), dopamine receptor-1 (Drd1) and dopamine receptor-2 (Drd2) (Spearman *ρ* Drd1; Drd2 = 0.38; 0.37; *P* < 0.0001, Bonferroni-corrected) as well as serotonergic receptor-1 (Htr1) and serotonergic receptor-3 (Hrt3) (Spearman *ρ* Hrt1; Hrt3 = 0.47; 0.30; *P* < 0.0001, Bonferroni-corrected). Interestingly, the 5-Hz tonic stimulation showed remarkable similarities with previously reported brain responses from chemogenetic activation of the LC, suggesting a convergence of the two experimental approaches^23^ (Spearman *ρ* = 0.33; *P* < 0.001, Bonferroni-corrected) (Supplementary Table 1). In addition to the α1A and Adrb relationship, burst activation maps at 15 Hz also correlated with spatial maps of the Drd2 (Spearman *ρ* = 0.30; *P* < 0.0001, Bonferroni-corrected) (Supplementary Table 1). No other meaningful association values emerged for other receptors or receptor subtypes (Supplementary Table 1). These findings provide insight into the underlying biological substrate that connects changes in LC activity to changes in forebrain activity and reconcile the results of previous studies on whole-brain effects of LC–NA stimulation^23,24^.

### LC–NA enhances network integration along a cortical gradient

Spatially coordinated, temporally sustained patterns of neural activity give rise to brain activity patterns that can change discretely or continuously, with or without external stimuli^45^. Earlier work based on human neuroimaging data suggested that LC activation could act as a facilitator of such dynamic changes^21^. Additionally, previous research also linked noradrenergic activity to enhanced system integration^21^, arguing that the LC–NA system is uniquely positioned to create a more integrated network topology^1,21^. Our optogenetic-fMRI approach enabled us to directly test this hypothesis. To this end, we quantified the distinct effects of optogenetically evoked tonic and burst-like LC firing on cortical and thalamic dynamics using topological analyses. Indeed, our data showed a significant increase in mean cortical participation coefficient (PC), a measure of network integration (Fig. 4g), from prestimulation to stimulation (Fig. 4h). Furthermore, 5-Hz tonic LC stimulation resulted in a significantly different mean cortical PC compared to sham stimulation (Fig. 4h).

We further conducted a region-wise analysis and plotted the delta of the PC against the hierarchical functional organization of cortical areas in mice. These results showed that a 3-Hz tonic stimulation pattern segregated the transmodal regions from primary somatosensory regions relative to a 15-Hz burst-like pattern (Extended Data Fig. 4e). In contrast, no regional relationship was observed between the 3-Hz and 5-Hz tonic stimulation intensities (Extended Data Fig. 4f). This suggests that changes in region modularity are consistent between the two tonic stimulations. Furthermore, a 5-Hz tonic intensity uniformly increased integration among cortical regions, as shown by the uniform change in PC, alluding to an additive effect of LC stimulation power on network integration.

Together, our findings show that LC stimulation has an influence on the system-level configuration of brain networks in vivo, with varying cortical and thalamic responses depending on the intensity and pattern of LC activation. This is noteworthy because it is an empirical demonstration that LC–NA firing patterns and intensity causally impact network dynamics.

## Discussion

Neuromodulation is one of the key processes that endows the brain’s relatively static structural architecture with flexibility, making it possible to support the malleable neural dynamics required for adaptive behavior^46^. As part of the neuromodulatory ascending arousal system, the LC–NA system is well placed to implement this function^4,21^. Previous animal and human studies hinted that high-tonic LC activity can rapidly reconfigure functional large-scale network architecture to facilitate coordination between otherwise segregated regions^1,4,15,23^. In this study, we addressed the question whether variations in the firing pattern of LC, as well as firing intensity, differentially impact brain dynamics. We used a combined optogenetic-fMRI approach to test levels of tonic and burst-like LC firing and visualize their effects on brain-wide activity in the anesthetized mouse. Across firing patterns and intensities, we report measurably different levels of NA release that translate to distinct BOLD signals and dynamic activity patterns in the forebrain. This is critical because it emphasizes the need to carefully consider the stimulation paradigms when assessing the impact of LC activity on brain function^46,47^. We also observed that when LC activity changes from a tonic to a burst-like pattern, downstream activation shifts in favor of sensory processing. This shift is accompanied by functional segregation of higher-order associate regions from somatosensory ones. These findings are important because they provide an explanation of how LC activity patterns and NA release can trigger adaptable network responses to continuously changing environmental demands^3,23,24^.

### LC–NA modulation dynamics and contributions in BOLD fMRI

The neuromodulatory and sympathetic effects of the LC–NA system may dissociate the conventional coupling between neuronal and vascular activity and thus change the typical functional hyperemic response^24,48^. For this reason, the interpretability of LC–NA BOLD-derived fMRI data is inherently elusive, as demonstrated by the observed biphasic BOLD signals in response to optogenetic LC–NA stimulation. Several studies hinted that a functional decrease in blood oxygenation can correspond to neuronal inhibition and constriction of arterioles initiated by neuronal release of neurotransmitters and neuropeptides associated with functional inhibition^34,49,50^. Using electrophysiology, we showed a time-locked increase in inhibitory neuronal activity in a downstream, subcortical target of LC. A recent fMRI study linked the activity of parvalbumin (PV) INTs to a transiently decreased hemodynamic response and vasoconstriction via suppression of excitatory neurons^35^. This was followed by a delayed functional hyperemia and vasodilation, probably driven by a PV-induced release of the vasoactive neuropeptide substance P (SP). Importantly, emotional stressors, commonly associated with increased LC–NA activity^28^, have been shown to affect SP signaling^51^. Our optogenetic LC–NA stimulation may have caused downstream PV INTs to suppress excitatory neurons, manifesting as a decrease in BOLD signal, while simultaneous SP efflux evoked a delayed vasodilation, manifesting as a slow increase in BOLD. These are testable hypotheses that should be addressed in future studies, particularly in light of studies documenting diverse neurochemical and cell type-specific contributions to the BOLD signal^52,53^. A comprehensive examination of the recent literature underscores the diverse impact of several neurotransmitters such as glutamate, γ-aminobutyric acid, acetylcholine, dopamine and neuropeptides on hemodynamics across different brain regions^53^. These effects are attributed to regional variations in cell types and receptor expression levels. Pharmacological or circuit approaches are available to explore these mechanisms, yet a detailed investigation of this approach was outside the scope of the present study.

Furthermore, the astrocytic activity driven by LC–NA may serve as an additional driver for delayed, positive BOLD signals^39,54^. Evoked or intrinsic BOLD responses demonstrate a correlation with heightened astrocytic activity, which may present as positive or negative depending on the brain’s state^39^. Other optical imaging studies likewise observed the initiation of induced Ca^2+^ signals in astrocytes in vivo, with a latency of a few seconds compared to the neuronal responses^55^. In this study, we showed robust increases in astrocytic Ca^2+^ activity on optogenetic LC stimulation that are reminiscent of our recorded NA release dynamics. Notably, we observed a strong correlation of fMRI signals with the Adra1a subtype, which is prominently expressed in astrocytes and known to be a prime target of NA^56^. With sustained LC activation and NA release, it is conceivable that astrocytic recruitment and local metabolic demand increase, leading to a gradual rise in tissue oxygenation that is reflected in an increase in BOLD signal^54^. On a related note, the diverse effects of LC–NA on downstream BOLD deflections are probably carried over to the stimulus offset period. ROIs consistently showed a similar but inverted time course (that is, overshoot or rebound) after the stimulation, suggesting sustained local neuromodulatory effects or possibly local measures of ‘gain control’ in response to a dynamic input modulation^21^. By considering both factors in our GLM model, we estimated the global influence of LC–NA stimulation patterns in a systematic manner and found that downstream activity patterns probably arise because of combined neural and vascular responses, reflecting the differential effects of the LC–NA system on fMRI signals. However, it is important to exercise caution when assessing the generalizability of any model across all brain regions. Acknowledging the diversity in cell type compositions across different areas, we recognize the need for careful consideration and perhaps additional validation methods to aid in LC–NA fMRI signal interpretation.

Overall, the complexity of putative neurochemical and cell type-specific interactions with the LC–NA system and its role in shaping brain hemodynamics is evident. This complexity underscores the necessity for continued research aimed at unraveling the mechanisms governing local vascular tone. Improved understanding in this area holds promise for enhancing the interpretation of fMRI data and guiding research into disease states characterized by distinct neurochemical signaling patterns.

### Hierarchical brain activity and LC–NA dynamic gain control

Intense or otherwise motivationally salient stimuli have been shown to evoke burst-like LC activity^13^. While tonic LC background activity continually modulates arousal, burst-like firing is believed to serve as a ‘wakeup call’ to facilitate increased task engagement and performance by differentially affecting brain activity^15,16^. In line with this idea, we showed that intensity-matched tonic and burst-like stimulation of LC noradrenergic neurons evoke divergent and dynamic patterns of brain activity, which hinge on their distinct NA release profiles. While burst-like stimulation recruits areas of the SSCtx, intermediate tonic stimulation engages the medial prefrontal and hippocampal regions, a distribution that aligns along a hierarchy of increasing functional integration^43^. Regions at the bottom of the hierarchy are linked to the processing of sensory input at a more fundamental level, whereas areas at the top of the hierarchy are often linked to an integrative role and processing of more sophisticated and abstract information^43^. This architecture allows for more effective information processing and greater flexibility to accommodate changes in environmental conditions^43,57^. However, what drives such a value ranking at the level of brain activity is less well understood. Our findings suggest that tonic and burst-like LC firing could act as dynamic gain controllers along this hierarchical axis, dynamically reassigning the brain’s resources according to situational demands.

More specifically, burst-like firing—typically related to salient stimuli that require sensory reorientation^2,6,26^—preferentially activates ‘lower-level’ sensory processing, whereas tonic firing—related to sustained attention and task engagement^2,6,25,26^—activates ‘higher regions’ tasked with more complex and abstract information processing. Importantly, these findings are further reflected in a cortical network segregation, where moderate tonic stimulation segregates transmodal regions from unimodal ones in contrast to a burst-like LC firing pattern. Our interpretation posits that the LC adopts a tonic firing rate during tasks requiring heightened focus, involving the engagement of transmodal regions such as frontal areas. Conversely, in situations necessitating increased alertness to environmental changes, the LC transitions to a burst-like firing mode, engaging sensory areas and thus facilitating adaptive behavioral responses. This dynamic modulation by the LC facilitates adaptive behavioral responses. Future avenues of investigation should explore the impact of stimuli-mediated brain responses in both the presence and absence of LC stimuli. It could also be particularly insightful to study this relationship in more naturalistic scenarios where awake animals are recorded concurrently with LC calcium activity. Such an approach could provide a more comprehensive understanding of how LC activity interacts with and influences broader neural dynamics during several cognitive and perceptual tasks in real-world contexts.

### Limitations

We acknowledge that unilateral and unimodal optogenetic stimulation sustained over 30 s—particularly with a burst-like pattern—does not represent a physiological mode of operation of the LC–NA system. In fact, the exact firing patterns of LC neurons in response to several environmental challenges, such as stressful stimuli, are unknown because of difficulties in recording large numbers of LC neurons, particularly in freely moving mice. The highest density recordings in anesthetized rats to date suggest highly variable firing of LC neurons, where only a fraction (15%) displayed synchronous firing associated with phasic discharge, even in response to a noxious foot shock^58^. However, it is conceivable that this number might increase in vivo in a scenario where multimodal stress signals converge and accumulate over minutes or hours. In vivo, firing in a new environment showed prominent burst activity in the range of 15–28 Hz sustained over several minutes^11^. Thus, a challenge for future work will be to decipher the natural firing properties of LC neurons over longer timescales and in response to various situational demands (sustained attention, salient stimuli, novelty, stress), and subsequently refine stimulation protocols to closely resemble (or even replay) naturally occurring LC activity patterns.

Furthermore, it is important to consider the potential impact of disparate consciousness states of mice during experiments on the results of our optogenetic stimulation (that is, anesthesia during fMRI data acquisition, sedation during pupillometry and fiber photometry recordings, awake during electrophysiological recordings). Particularly during fMRI measurements, even with careful titration, anesthesia dampens spontaneous LC background activity^59^, which may affect the observed effects of LC firing patterns and intensities at a whole-brain level. In future studies conducted in the awake state, these activity patterns may be attenuated because of the interplay between the LC–NA system and other neuromodulators that contribute to arousal and are more active during wakefulness^60^. Similarly, it will be worthwhile to assess the extent of downstream dopamine co-release—a feature of the LC–NA system that is linked to its activity patterns^11,23^—and their effects on whole-brain activity. Given the significant correlation of NA-dependent activity at 5-Hz LC stimulation and Drd1 distribution maps, it is conceivable that at least some of the observed BOLD patterns at a high-tonic LC–NA output are affected by dopaminergic modulation or an interaction between dopamine and NA. In addition, we cannot exclude potential influences of the contralateral hemisphere on our fMRI signals. To this point, conveyed activity in the contralateral LC during 5-Hz stimulation might have contributed to shaping the distinct whole-brain activation profiles of the higher tonic dataset. Finally, our work could not address the modular organization of the LC into cellular ensembles^6^, as our stimulation will uniformly drive LC activity irrespective of afferent and efferent projections.

## Conclusions

In this work, we discovered that shifts in LC firing patterns evoke dynamic changes in brain activity and network reconfigurations at the systems level. Our data show that tonic and burst-like LC stimulation influence the brain’s processing disposition to prioritize associative information over somatosensory information and vice versa. Additionally, we demonstrated how both tonic and burst-like LC firing patterns are related to dynamic modulation of cortical network topology, presenting a view of a potential role of the LC–NA system in gating brain state transition. Together, our results provide new insight into how the LC–NA system continuously and adaptively modulates brain dynamics to support cognitive functions and ongoing behaviors.

## Methods

### Mouse lines

All animal procedures were conducted in accordance with the Swiss federal guidelines for the use of animals in research and approved by the Cantonal Veterinary Office of Zürich. Heterozygous C57BL/6-Tg(*Dbh*^iCre^)1Gsc (*Dbh*^iCre^) mice^62^ were kept in standard housing on a 12 h light–dark cycle, with housing temperatures between 18 °C and 23 °C and 40–60% humidity. Food and water were provided ad libitum.

### Stereotaxic surgery

Stereotaxic surgery was performed on 2–3-months-old *Dbh*^iCre^ mice under 2% isoflurane anesthesia with a subcutaneous dose of 5 mg kg^−1^ meloxicam and a local anesthetic (Emla cream; 5% lidocaine, 5% prilocaine). Animals were placed into a stereotaxic apparatus and their skulls exposed. Bregma was located and the skull placement corrected for tilt and scaling. For virus delivery and optical fiber implantation in the right LC, a small hole was drilled at anterior-posterior (AP) −5.4 mm and mediolateral (ML) −0.9 mm, relative to bregma. For pupillometry (*n* male mice = 13, *n* female mice = 12) and optogenetic-fMRI recordings (*n* male mice = 10, *n* female mice = 12), mice were then injected unilaterally (coordinates: AP −5.4 mm, ML −0.9 mm, dorsoventral (DV) −3.8 mm) with 1 µl of an AAV construct carrying the optogenetic actuator ChR2 (ssAAV-5/2-hEF1α-dlox-hChR2(H134R)_EYFP(rev)-dlox-WPRE-hGHp(A); 4.7 × 10^12^ vg ml^−1^; Viral Vector Facility (VVF), Neuroscience Center Zürich) using a pneumatic injector (IM-11-2, Narishige) and calibrated microcapillaries (cat. no. P0549, Sigma-Aldrich). For fiber photometry recordings, mice were injected unilaterally in the LC (coordinates: AP −5.4 mm, ML −0.9 mm, DV −3.8 mm), with 1 µl of an AAV construct carrying the optogenetic actuator ChrimsonR (ssAAV-5/2-hEF1α/hTLV1-dlox-ChrimsonR_tdTomato(rev)-dlox-WPRE-bGHp(A); 4.7 × 10^12^ vg ml^−1^; VVF, Neuroscience Center Zürich). Additionally, mice were injected with 0.2 µl of the genetically encoded NA sensor GRAB_NE1m_ (ssAAV-9/2-hSyn1-GRAB(NE1m)-WPRE-hGHp(A); 5.5 × 10^12^ vg ml^−1^; VVF, Neuroscience Center Zürich; *n* male mice = 3, *n* female mice = 9) or the astrocytic Ca^2+^ sensor GFAP-GCaMP6s (ssAAV-9/2-hGFAP-hHBbI/E-GCaMP6s-bGHp(A); 5 × 10^12^ vg ml^−1^; VVF, Neuroscience Center Zürich; *n* male mice = 2, *n* female mice = 2) in the ipsilateral hippocampus (coordinates: AP −3.2 mm, ML −3.3 mm, DV −3.8 mm). Subsequently, an optical fiber was implanted at 200 μm superior to the injection coordinates (for pupillometry and optogenetic-fMRI: low profile, 90° 200 μm, numerical aperture = 0.66; Doric Lenses; for fiber photometry: 200 µm, NA = 0.37; Neurophotometrics). Optical fibers were glued to the skull using a bonding agent (Etch glue, Heraeus Kulzer) and an ultraviolet-curable dental composite (Permaplast LH Flow; M+W Dental); stitches were used as required. The health of the animals was evaluated using postoperative checks over the course of 3 consecutive days and 5 mg kg^−1^ meloxicam administered subcutaneously if needed.

### Pupil recordings

For pupil recordings, a Raspberry Pi NoIR Camera Module V2 night vision camera, an infrared light source (Pi Supply Bright Pi, Bright White and IR camera light for Raspberry Pi) and a Raspberry Pi 3 Model B (Raspberry Pi Foundation) were used. Experimental procedures in all animals followed the guidelines detailed in ref. ^63^. Briefly, mice were anesthetized in an induction chamber with isoflurane in a 1:4 O_2_ to air mixture (4% induction, 2% maintenance). Anesthesia levels were maintained at 2% isoflurane throughout the recordings via a breathing mask to dampen and silence ongoing LC background activity^59^. The eye ipsilateral to the stimulated LC (right eye) was recorded and a 2-min baseline recording preceded the different stimulation paradigms. Stimulation patterns lasted 10 s and included (1) a 3-Hz (473 nm, 10 ms pulse width, 10 mW laser power), (2) a 5-Hz (473 nm, 10 ms pulse width, 10 mW laser power), (3) a 15-Hz burst stimulation (three pulses per second, 473 nm, 10 ms pulse width, 10 mW laser power) and (4) a 5-Hz sham stimulation (635 nm, 10 ms pulse width, 10 mW laser power). A red-light instead of a blue-light sham condition was chosen for this study to keep the total number of experimental animals to a minimum. Notably and like our red-light sham stimulation, a blue-light sham condition did not induce residual pupillary responses as shown in ref. ^63^. All stimulations were followed by 90 s of no laser light delivery. One animal (male) was excluded because of the absence of a pupil response.

### Fiber photometry

The green fluorescence signal from the NA sensor GRAB_NE1m_ or the astrocytic Ca^2+^ sensor GFAP-GCaMP6s was recorded using a commercially available photometry system (Model FP3002, Neurophotometrics) controlled using the open-source software Bonsai (v.2.6.2). Throughout the recording session, mice were lightly anesthetized (4% isoflurane during induction, 2% during maintenance) and the fiber implanted in the mouse brain was attached to a prebleached recording patch cord (200 μm, numerical aperture = 0.39, Doric Lenses). Two LEDs were used to deliver an interleaved excitation light: a 470-nm LED to record an NA/Ca^2+^-dependent fluorescence signal (F^470^) and a 415-nm LED for an NA/Ca^2+^-independent control fluorescence signal (F^415^). The recording rate was set at 120 Hz for both LEDs allowing 60 Hz for each channel individually. The excitation power at the fiber tip was set to 25–35 μW. For short-term, single-stimulation photometry recordings, the LC was stimulated using 3-Hz tonic, 5-Hz tonic and 15-Hz burst laser pulses in a randomized order during the same session (635 nm laser (CNI laser) at 5 mW output power). Long-term recordings to assess NA/Ca^2+^ responses for repeated stimulations were conducted in a randomized order on separate days.

### In vivo electrophysiological and fiber photometry recordings

For the in vivo electrophysiological recordings combined with fiber photometry, 8–9-weeks-old male NET-cre::Ai148 (GCaMP6f) mice (provided by S. Tonegawa) were implanted with a 400-µm optic fiber (FP400RT) unilaterally 100 µm above the LC. After 2 weeks of recovery, mice underwent 3–4 head fixation habituation sessions starting with 15 min and gradually increasing to 25 min; 3–4 weeks after surgery, craniotomies were performed to provide access to the dorsal hippocampus CA1. Craniotomies were covered with silicon sealant to protect the brain surface from mechanical impact, dehydration and light exposure before the silicon probe recording sessions. For the electrophysiological recordings, each mouse was gradually habituated to head fixation over multiple sessions and was allowed to run freely on a horizontal wheel. A 4-shank, 128-channel silicon microprobe (128DN; four shanks, 150-mm shank spacing, 25-mm channel spacing, 100-mm^2^ electrode area, 7 × 65 × 23 mm^3^ shank dimensions; provided S. Masmanidis, UCLA)^64^ or Neuropixels probe was inserted at a depth of approximately 2 mm or 4 mm correspondingly, with an insertion speed of 100 µm min^−1^. Before each recording session, the silicon probe recording sites were electroplated in a poly(3,4-ethylenedioxythiophene) solution to an impedance of 100 kOhm (for passive silicon probes). Silicon probes were connected to an RHD2000 chip-based 128-channel amplifier board (Intan Technologies) or the Neuropixels headstage. Broadband (0.1 Hz to 7.5 kHz) signals were acquired at 30 kHz. Signals were digitized at 16-bit and transmitted to an OpenEphys recording controller. Photometry recordings were performed with unilaterally implanted, custom-made optic fiber connectors (FP400URT, numerical aperture = 0.50, Ø 400 μm; Thorlabs). A modified Doric fiber photometry system was used to perform the recordings (Doric Neuroscience Studio v.6.1). Two different excitation wavelengths were used (465 nm for HC-dependent GCaMP6f activity and 405 nm to record an isosbestic, Ca^2+^-independent reference signal that served to correct for photobleaching and movement-related artifacts).

### Optogenetic-fMRI recording

A total of 87 fMRI scans were acquired with adult, heterozygous male (*n* = 22) and female (*n* = 7) *Dbh*^iCre^ mice. Six fMRI scans were excluded because of fMRI coil-related artifacts, leaving a final dataset of 81 scans.

#### Animal preparation

For the fMRI scans, mice were anesthetized in a gas chamber for 4 min with 4% isoflurane in 1:4 O_2_ to air mixture. Animals were endotracheally intubated and their tail vein cannulated while being kept under anesthesia with 2% isoflurane. During preparation, animal temperature was kept at 37 °C using a heating pad (Harvard Apparatus). Once intubated and cannulated, mice were head-fixed with ear bars and connected to a small animal ventilator (CWE, Ardmore) on an MRI-compatible support. Ventilation was set to 80 breaths per minute, with 1.8 ml min^−1^ flow with isoflurane at 2%. A bolus injection of a muscle relaxant (pancuronium bromide, 0.25 mg kg^−1^) was delivered via the cannulated vein and isoflurane was reduced to 1.5%. A fiberoptic patch cord (Thorlabs) connected to a custom-made diode-pumped solid-state laser (CNI laser) was tethered to the optical fiber implant via an MRI-compatible connector. Continuous infusion of pancuronium bromide (0.25 mg kg h^−1^) started 5 min after the initial bolus injection. Isoflurane was reduced to 1.1%. A hot water circulation bed kept the temperature of the animal constant throughout the entire measurement (35 °C). Additionally, body temperature was monitored using a rectal thermometer probe. After collection of functional and anatomical MRI scans, the continuous injection and isoflurane flow was stopped. Animals remained connected to the ventilator until independent breathing could be assured and then transferred to a heating chamber for further recovery.

#### Data acquisition

Data were acquired in a 7T Bruker BioSpec scanner equipped with a Pharmascan magnet and a high signal-to-noise ratio dedicated mouse brain quadrature T/R cryogenic coil (Bruker BioSpin) (gradient: 7T, 16-cm bore size, 9-cm head gradient insert, maximum gradient 770 mT m^−2^, rise time 110 µs, AVANCE III electronics). Standard adjustments included calibration of the reference frequency power and shim gradients using MAPSHIM (Paravision v.6.1). For anatomical assessment, a T1-weighted image was acquired via a FLASH sequence with an in-plane resolution of 0.05 × 0.02 mm^2^, an echo time (TE) of 3.51 ms and a repetition time (TR) of 522 ms. For the functional scans, a standard gradient-echo echo-planar imaging sequence (GE-EPI, TR = 1 s, TE = 15 ms, in-plane resolution = 0.22 × 0.2 mm^2^, number of slices = 20, slice thickness = 0.4 mm, slice gap = 0.1 mm) was applied to acquire 1,440 volumes in 24 min. To reduce acquisition time, we used a partial Fourier with an acceleration of 1.2 in the phase domain.

#### Optogenetic stimulation

The intensity and firing pattern-dependent whole-brain activity of ChR2-EYFP-expressing *Dbh*^iCre^ mice was recorded in separate functional scans. Over the course of 4 weeks, mice were randomly assigned to the 3 Hz, 5 Hz, 15 Hz and sham optogenetic stimulation protocols (pulse width = 10 ms). Using a randomized block design, a single mouse underwent a maximum of one scanning session and optogenetic protocol per week to allow for sufficient isoflurane washout and avoid any potential ‘spill-over’ effects on brain activation patterns between stimulation patterns, respectively. This way, over the course of 4 weeks, an experimental animal underwent a maximum of four scanning sessions. Functional scans consisted of an 8-min prestimulation baseline, an 8-min optogenetic stimulation phase and an 8-min poststimulation baseline. For the 3-Hz and 5-Hz tonic optogenetic stimulation, trains of 473 nm laser pulses were delivered for 30 s at 10 mW laser power above the targeted site, followed by 30 s of no laser light delivery. This stimulation paradigm was repeated over the course of 8 min. For LC burst stimulation, a burst of three pulses at 15 Hz (10 mW and 473 nm) was delivered every second for 30 s, followed by 30 s of no laser light delivery. The stimulation was repeated over the course of 8 min. For the sham optogenetic stimulation, trains of 635 nm laser pulses were delivered at 3–5 Hz for 30 s and 10 mW laser power above the targeted site, followed by 30 s of no laser light delivery. The stimulation was repeated over the course of 8 min. Control measures against optogenetic light artifacts in the dark MRI scanner environment were taken as in ref. ^65^. Briefly, a continuously lit LED light source and a continuously lit blue laser light source were placed on the MRI cradle in the vicinity of the animal’s head to mask any potential light spill leading to visual artifacts during optogenetic laser pulse deliveries. Mice were randomly subjected to any of the four optogenetic stimulation protocols.

### Immunohistochemistry

The hindbrain, including the LC, was fixed in 4% PFA for 2 h, cryoprotected in a sucrose solution and frozen in mounting medium. The hindbrain was then cut into 40-µm sections and stained in primary antibody solution containing 0.2% Triton X-100, and 2% normal goat serum in PBS at 4 °C under continuous agitation over two nights. Afterwards, sections were washed three times in PBS for 10 min per cycle and then transferred into secondary antibody solution containing 2% normal goat serum in PBS. After three more PBS washes, the sections were mounted onto glass slides (Menzel-Glaser SUPERFROST PLUS, Thermo Fisher Scientific), air-dried and cover-slipped with Dako fluorescence mounting medium (Agilent Technologies). Primary antibodies included: mouse anti-TH (1:1,000 dilution, cat. no. 22941, Immunostar); chicken anti-GFP (1:1,000 dilution, cat. no. ab13970, Abcam); and rabbit anti-cFOS (1:5,000 dilution, cat. no. 226 003, Synaptic Systems). Secondary antibodies included: donkey anti-mouse Alexa Fluor 647 (1:300 dilution, cat. no. A-31571, Thermo Fisher Scientific); goat anti-chicken Alexa Fluor 488 (1:300 dilution, cat. no. A-11039, Thermo Fischer Scientific); and goat anti-rabbit Alexa Fluor 546 (1:300 dilution, cat. no. A11035, Thermo Fisher Scientific). Microscopy images were acquired in a confocal laser-scanning microscope (LSM 880, ZEISS). Images of the LC were acquired in a separate cohort to evaluate LC targeting (*n DBH*^iCre^ male = 11) using a ×10 or ×20 objective.

### Quantification and statistical analysis

#### Quantification

Pupil diameter was measured using the motion tracking software DeepLabCut^66,67^. The web-based pupillometry app reported in ref. ^63^ was used for visualization and analysis. Measurements after 3-Hz, 5-Hz and 15-Hz optogenetic stimulation were normalized to the 10-s baseline preceding laser light delivery and were statistically analyzed as in ref. ^63^.

#### Fiber photometry

Analysis of the raw photometry data was performed using a custom-written MATLAB script. First, to filter high-frequency noise (above 1 Hz), the lowpass filter function was applied to both recorded signals (F^470^ and F^415^). Next, to correct for photobleaching of the fluorescence signal, the baseline fluorescence *F*^415^_baseline fit_ was calculated as a linear fit applied to the filtered F^415^ to F^470^ signals during the baseline 5-s window preceding each LC stimulation. Finally, the signal of the NA sensor was expressed as a percentage change in fluorescence: Δ*F/F* = 100 × (*F*^470^(*t*) − *F*^415^_baseline fit_(*t*))/*F*^415^_baseline fit_(*t*), where *F*^470^(*t*) signifies the filtered fluorescence value at each time point *t* across the recording and *F*^415^_baseline fit_(*t*) denotes the value of the fitted 415-nm signal at the time point *t*. The final Δ*F/F* signal was smoothed with a 10–50-point moving mean filter. To capture the difference in bleaching dynamics for the F^470^ and F^415^ of the GRABNE1m sensor over the long-term photometry recording, the 405 and 465-nm excited signals were individually fitted with a third-degree polynomial function using the MATLAB polyfit function. Fitting parameters were calculated over a time window including a 1-min interval before the first stimulation onset and a 1-min interval at the end of the recording (4 min after the offset of the last stimulation). Each signal was then divided by its fit to correct for bleaching and normalize each signal. Finally, the ∆*F/F* for each time point was calculated as the difference between the bleaching-corrected, normalized signals excited at 405 and 465 nm: Δ*F/F* = 100 × ((Δ*F/F*)^470^_normalized_ − (Δ*F/F*)^415^_normalized_). Similarly, the final Δ*F/F* signal was smoothed with a 100-point moving mean filter.

#### In vivo electrophysiology and fiber photometry analysis

Raw electrophysiology data were processed to detect spikes and extract single-unit activity. Briefly, wideband signals were bandpass-filtered (0.6–6 kHz), spatially whitened across channels and thresholded for the isolation of putative spikes. Clustering was performed using template matching implemented in Kilosort3 (ref. ^68^); the computed cluster metrics were used to preselect units for later manual curation using custom-written software. Single units were classified as putative excitatory principal neurons and fast-spiking INTs based on their extracellular waveforms (Fig. 3c). After classification, the response of the principal neurons and INTs was used to evaluate the mean response of the respective populations. For response characterization, the spike rates were calculated in 100-ms bins. Each unit’s activity was normalized to the average firing rate in the 5 s before the trigger event (intrinsic GCaMP6f response).

Photometry data were preprocessed and analyzed using custom programs written in MATLAB (v.2017b, MathWorks). Data with obvious motion artifacts in the isosbestic channel were discarded. Demodulated raw Ca^2+^ traces were downsampled to 1 kHz and then detrended using a low-cut filter (Gaussian, cutoff of 2–4 min) to correct for slow drift of the baseline signal due to bleaching. Filtered traces were *z*-scored by the mean and s.e. of the entire trace.

#### fMRI data analysis

##### ROI analysis

ROIs were chosen anatomically using the Mouse Brain Allen Common Coordinate Framework v.3 (CCv3) and according to the hierarchical gradient of the mouse cortex as outlined in ref. ^43^. For the latter, a total of 38 cortical ROIs were extracted: ACAd; ACAv; Ald; Alp; Alv; AUDd; AUDp; AUDv; ECT; FRP; GU; ILA; MOp; MOs; ORBI; ORBm; ORBvl; PERI; PL; PTLp; RSPagl; RSPd; RSPv; SSp-bfd; SSp-ll; SSp-m; SSp-n; SSp-t; SSp-ul; SSs; TEa; VISal; VISam; VISC; VISl; VISp; VISpl; VISpm. For visualization purposes, six additional cortical and subcortical ROIs were extracted: mPFC, CPu, SSCtx, HC, TH and AMY. Mean time series for selected anatomical ROIs were extracted and corrected for baseline.

##### GLM statistical mapping

Preprocessing and functional data analysis was carried out using FSL FEAT (v.5.92, www.fmrib.ox.ac.uk/fsl) and in-house MATLAB scripts. Preprocessing included the following steps: (1) B1 field inhomogeneity correction using the N4 algorithm as provided in the software package Advanced Normalization Tools (ANTs) v.2.1.0 (ref. ^69^); (2) discarding the first ten measurements to achieve steady-state excitation; (3) high-pass filtering (with a cutoff of 90 s) and motion correction using MCFLIRT. Because of the small size of the optogenetically targeted LC and downstream activated regions, no smoothing kernel was applied. To account for potential alignment artifacts due to the implanted optical fiber, two study-specific templates based on all mean echo-planar images (EPIs) and T1-weighted anatomical images were created using ANTs (http://stnava.github.io/ANTs/). Registration was carried out first for the study-specific EPI template and then for the T1-weighted template using FLIRT and FNIRT. Time series statistical analysis was carried out using FILM and standard motion parameter correction. To fully capture the biphasic nature of the BOLD responses evoked by LC stimulation, we implemented a GLM that incorporated two main regressors: one representing the ON–OFF laser stimulation design and the other comprising an NA release trace computed from an average of the 3-Hz tonic, 15-Hz burst and 5-Hz tonic fiber photometry traces. The sum of these regressors (that is, the logical ‘AND’) was then convolved with a standard double-gamma HRF. After the application of standard motion parameters (MCFLIRT), group-level analysis was conducted using a fixed-effects analysis. Statistical analysis was done comparing whole-brain group-level activity maps for each stimulation protocol against the sham condition and across stimulation types. Alternatively, individual regressors were used to qualitatively assess their impact on the evoked BOLD signal under the specific conditions. All *z*-statistic images underwent thresholding using the clusters identified by *z* > 3.1, with a family-wise error-corrected cluster significance threshold of *P* = 0.05 applied to the suprathreshold clusters^70,71^.

For visualization purposes, averaged group and group contrast activation maps were normalized to a high-resolution Allen Brain Institute mouse brain atlas using affine and nonlinear greedy transformations. To allow for detailed assessment of incurred distortion, averaged group activation maps were additionally mapped onto the study-specific GE-EPI template.

##### Time-resolved network analyses

To inspect functional connectivity between the aforementioned 38 cortical ROIs, we used the Louvain modularity algorithm from the Brain Connectivity Toolbox on the functional connectivity edge weights to estimate community structure^72^. The stability of the gamma parameter was determined through a permutation process and defined as 1.3 for all analyses. We used the partition from the modularity algorithm to estimate the PC from unthresholded, weighted and signed connectivity matrices. The PC quantifies the extent to which a region connects across all modules (that is, between-module strength). The PC is close to 1 if its connections are uniformly distributed among all the modules and 0 if all of its links are within its own module. The mean and individual region PC scores were then compared across animals for the different stimulation types, after first correcting for the baseline associations observed during prestimulation ‘resting-state’ epochs.

#### Statistics and reproducibility

The statistical details for every experiment are provided in the figure legends, where ‘*n*’ represents the number of animals per group. No statistical methods were used to predetermine sample sizes but our sample sizes are similar to those reported in previous publications^65,73^. Data distribution was assumed to be normal but this was not formally tested. Statistical significance was defined as *P* < 0.05. Data were excluded in the absence of pupillary responses or because of fMRI coil-related artifacts. The experiments were randomized; however, investigators were not blinded to allocation during the experiments and outcome assessment.

### Reporting summary

Further information on research design is available in the Nature Portfolio Reporting Summary linked to this article.

## Online content

Any methods, additional references, Nature Portfolio reporting summaries, source data, extended data, supplementary information, acknowledgements, peer review information; details of author contributions and competing interests; and statements of data and code availability are available at 10.1038/s41593-024-01755-8.

## Supplementary information

Supplementary Information Supplementary Table 1.

Reporting Summary

## Data Availability

Data supporting the findings of this study are available within the article and its supplementary information files. Raw MRI data are publicly available at https://zenodo.org/record/7064020#.Yyw2ri8Rpjl (ref. ^74^). Anatomical regions of the mouse brain were chosen based on the Mouse Brain Allen Common Coordinate Framework v.3 (https://connectivity.brain-map.org/static/referencedata#:~:text=The%20Allen%20Mouse%20Brain%20Common,using%20serial%20two%2Dphoton%20tomography).

## Code availability

Imaging data were analyzed using FSL FEAT v.5.92 (www.fmrib.ox.ac.uk/fsl) and Advanced Normalization Tools v.2.1.0 (http://stnava.github.io/ANTs/). Scripts for analyzing the PC can be accessed via GitHub at https://github.com/bmunn/IntegrationLCStim. Figures were created using BioRender.com and Adobe Illustrator.

## References

[1] Shine, J. M. Neuromodulatory influences on integration and segregation in the brain. *Trends Cogn. Sci.* **23**, 572–583 (2019). 31076192 10.1016/j.tics.2019.04.002

[2] Aston-Jones, G. & Cohen, J. D. Adaptive gain and the role of the locus coeruleus-norepinephrine system in optimal performance. *J. Comp. Neurol.* **493**, 99–110 (2005). 16254995 10.1002/cne.20723

[3] Bouret, S. & Sara, S. J. Network reset: a simplified overarching theory of locus coeruleus noradrenaline function. *Trends Neurosci.* **28**, 574–582 (2005). 16165227 10.1016/j.tins.2005.09.002

[4] Shine, J. M. et al. Human cognition involves the dynamic integration of neural activity and neuromodulatory systems. *Nat. Neurosci.* **22**, 289–296 (2019). 30664771 10.1038/s41593-018-0312-0

[5] Berridge, C. W. & Waterhouse, B. D. The locus coeruleus-noradrenergic system: modulation of behavioral state and state-dependent cognitive processes. *Brain Res. Brain Res. Rev.* **42**, 33–84 (2003). 12668290 10.1016/s0165-0173(03)00143-7

[6] Poe, G. R. et al. Locus coeruleus: a new look at the blue spot. *Nat. Rev. Neurosci.* **21**, 644–659 (2020). 32943779 10.1038/s41583-020-0360-9 PMC8991985

[7] Totah, N. K. B., Logothetis, N. K. & Eschenko, O. Noradrenergic ensemble-based modulation of cognition over multiple timescales. *Brain Res.* **1709**, 50–66 (2019). 30586547 10.1016/j.brainres.2018.12.031

[8] Bouret, S. & Sara, S. J. Reward expectation, orientation of attention and locus coeruleus-medial frontal cortex interplay during learning. *Eur. J. Neurosci.* **20**, 791–802 (2004). 15255989 10.1111/j.1460-9568.2004.03526.x

[9] Rajkowski, J., Majczynski, H., Clayton, E. & Aston-Jones, G. Activation of monkey locus coeruleus neurons varies with difficulty and performance in a target detection task. *J. Neurophysiol.* **92**, 361–371 (2004). 15028743 10.1152/jn.00673.2003

[10] Vankov, A., Hervé-Minvielle, A. & Sara, S. J. Response to novelty and its rapid habituation in locus coeruleus neurons of the freely exploring rat. *Eur. J. Neurosci.* **7**, 1180–1187 (1995). 7582091 10.1111/j.1460-9568.1995.tb01108.x

[11] Takeuchi, T. et al. Locus coeruleus and dopaminergic consolidation of everyday memory. *Nature* **537**, 357–362 (2016). 27602521 10.1038/nature19325 PMC5161591

[12] Clayton, E. C., Rajkowski, J., Cohen, J. D. & Astone-jones, G. Phasic activation of monkey locus ceruleus neurons by simple decisions in a forced-choice task. *J. Neurosci.* **24**, 9914–9920 (2004). 15525776 10.1523/JNEUROSCI.2446-04.2004 PMC6730226

[13] Aston-Jones, G. & Bloom, F. E. Nonrepinephrine-containing locus coeruleus neurons in behaving rats exhibit pronounced responses to non-noxious environmental stimuli. *J. Neurosci.* **1**, 887–900 (1981). 7346593 10.1523/JNEUROSCI.01-08-00887.1981 PMC6564231

[14] Mather, M., Clewett, D., Sakaki, M. & Harley, C. W. Norepinephrine ignites local hotspots of neuronal excitation: how arousal amplifies selectivity in perception and memory. *Behav. Brain Sci.* **39**, e200 (2016). 26126507 10.1017/S0140525X15000667 PMC5830137

[15] Unsworth, N. & Robison, M. K. A locus coeruleus-norepinephrine account of individual differences in working memory capacity and attention control. *Psychon. Bull. Rev.* **24**, 1282–1311 (2017). 28108977 10.3758/s13423-016-1220-5

[16] Sara, S. J. & Bouret, S. Orienting and reorienting: the locus coeruleus mediates cognition through arousal. *Neuron* **76**, 130–141 (2012). 23040811 10.1016/j.neuron.2012.09.011

[17] Carter, M. E. et al. Tuning arousal with optogenetic modulation of locus coeruleus neurons. *Nat. Neurosci.* **13**, 1526–1533 (2010). 21037585 10.1038/nn.2682 PMC3174240

[18] Devilbiss, D. M. Consequences of tuning network function by tonic and phasic locus coeruleus output and stress: regulating detection and discrimination of peripheral stimuli. *Brain Res.* **1709**, 16–27 (2019). 29908165 10.1016/j.brainres.2018.06.015

[19] Hermans, E. J., Henckens, M. J. A. G., Joëls, M. & Fernández, G. Dynamic adaptation of large-scale brain networks in response to acute stressors. *Trends Neurosci.* **37**, 304–314 (2014). 24766931 10.1016/j.tins.2014.03.006

[20] Hasenkamp, W., Wilson-Mendenhall, C. D., Duncan, E. & Barsalou, L. W. Mind wandering and attention during focused meditation: a fine-grained temporal analysis of fluctuating cognitive states. *Neuroimage* **59**, 750–760 (2012). 21782031 10.1016/j.neuroimage.2011.07.008

[21] Munn, B. R., Müller, E. J., Wainstein, G. & Shine, J. M. The ascending arousal system shapes neural dynamics to mediate awareness of cognitive states. *Nat. Commun.* **12**, 6016 (2021). 34650039 10.1038/s41467-021-26268-x PMC8516926

[22] Hermans, E. J. et al. Stress-related noradrenergic activity prompts large-scale neural network reconfiguration. *Science* **334**, 1151–1153 (2011). 22116887 10.1126/science.1209603

[23] Zerbi, V. et al. Rapid reconfiguration of the functional connectome after chemogenetic locus coeruleus activation. *Neuron* **103**, 702–718 (2019). 31227310 10.1016/j.neuron.2019.05.034

[24] Oyarzabal, E. A. et al. Chemogenetic stimulation of tonic locus coeruleus activity strengthens the default mode network. *Sci. Adv.* **8**, eabm9898 (2022). 35486721 10.1126/sciadv.abm9898 PMC9054017

[25] Usher, M., Cohen, J. D., Servan-Schreiber, D., Rajkowski, J. & Aston-Jones, G. The role of locus coeruleus in the regulation of cognitive performance. *Science* **283**, 549–554 (1999). 9915705 10.1126/science.283.5401.549

[26] Rajkowski, J., Kubiak, P. & Aston-Jones, G. Locus coeruleus activity in monkey: phasic and tonic changes are associated with altered vigilance. *Brain Res. Bull.* **35**, 607–616 (1994). 7859118 10.1016/0361-9230(94)90175-9

[27] Bari, A. et al. Differential attentional control mechanisms by two distinct noradrenergic coeruleo-frontal cortical pathways. *Proc. Natl Acad. Sci. USA* **117**, 29080–29089 (2020). 33139568 10.1073/pnas.2015635117 PMC7682591

[28] McCall, J. G. et al. CRH engagement of the locus coeruleus noradrenergic system mediates stress-induced anxiety. *Neuron* **87**, 605–620 (2015). 26212712 10.1016/j.neuron.2015.07.002 PMC4529361

[29] Robertson, S. D., Plummer, N. W., de Marchena, J. & Jensen, P. Developmental origins of central norepinephrine neuron diversity. *Nat. Neurosci.* **16**, 1016–1023 (2013). 23852112 10.1038/nn.3458 PMC4319358

[30] Berridge, C. W. & Abercrombie, E. D. Relationship between locus coeruleus discharge rates and rates of norepinephrine release within neocortex as assessed by in vivo microdialysis. *Neuroscience* **93**, 1263–1270 (1999). 10501450 10.1016/s0306-4522(99)00276-6

[31] Osorio-Forero, A. et al. Noradrenergic circuit control of non-REM sleep substates. *Curr. Biol.* **31**, 5009–5023 (2021). 34648731 10.1016/j.cub.2021.09.041

[32] Feng, J. et al. A genetically encoded fluorescent sensor for rapid and specific in vivo detection of norepinephrine. *Neuron* **102**, 745–761 (2019). 30922875 10.1016/j.neuron.2019.02.037 PMC6533151

[33] Karalis, N. & Sirota, A. Breathing coordinates cortico-hippocampal dynamics in mice during offline states. *Nat. Commun.* **13**, 467 (2022). 35075139 10.1038/s41467-022-28090-5 PMC8786964

[34] Devor, A. et al. Suppressed neuronal activity and concurrent arteriolar vasoconstriction may explain negative blood oxygenation level-dependent signal. *J. Neurosci.* **27**, 4452–4459 (2007). 17442830 10.1523/JNEUROSCI.0134-07.2007 PMC2680207

[35] Vo, T. T. et al. Parvalbumin interneuron activity drives fast inhibition-induced vasoconstriction followed by slow substance P-mediated vasodilation. *Proc. Natl Acad. Sci. USA* **120**, e2220777120 (2023). 37098063 10.1073/pnas.2220777120 PMC10161000

[36] Uhlirova, H. et al. Cell type specificity of neurovascular coupling in cerebral cortex. *eLife* **5**, e14315 (2016). 27244241 10.7554/eLife.14315 PMC4933561

[37] Rupprecht, P. et al. Centripetal integration of past events in hippocampal astrocytes regulated by locus coeruleus. *Nat. Neurosci.* **27**, 927–939 (2024). 38570661 10.1038/s41593-024-01612-8 PMC11089000

[38] Iadecola, C. & Nedergaard, M. Glial regulation of the cerebral microvasculature. *Nat. Neurosci.* **10**, 1369–1376 (2007). 17965657 10.1038/nn2003

[39] Wang, M., He, Y., Sejnowski, T. J. & Yu, X. Brain-state dependent astrocytic Ca^2+^ signals are coupled to both positive and negative BOLD-fMRI signals. *Proc. Natl Acad. Sci. USA* **115**, E1647–E1656 (2018). 29382752 10.1073/pnas.1711692115 PMC5816146

[40] Winship, I. R., Plaa, N. & Murphy, T. H. Rapid astrocyte calcium signals correlate with neuronal activity and onset of the hemodynamic response in vivo. *J. Neurosci.* **27**, 6268–6272 (2007). 17554000 10.1523/JNEUROSCI.4801-06.2007 PMC6672142

[41] Salgado, H. et al. Layer-specific noradrenergic modulation of inhibition in cortical layer II/III. *Cereb. Cortex* **21**, 212–221 (2011). 20466749 10.1093/cercor/bhq081 PMC3000571

[42] Rodenkirch, C., Liu, Y., Schriver, B. J. & Wang, Q. Locus coeruleus activation enhances thalamic feature selectivity via norepinephrine regulation of intrathalamic circuit dynamics. *Nat. Neurosci.* **22**, 120–133 (2019). 30559472 10.1038/s41593-018-0283-1 PMC6301066

[43] Fulcher, B. D., Murray, J. D., Zerbi, V. & Wang, X.-J. Multimodal gradients across mouse cortex. *Proc. Natl Acad. Sci. USA* **116**, 4689–4695 (2019). 30782826 10.1073/pnas.1814144116 PMC6410879

[44] Lein, E. S. et al. Genome-wide atlas of gene expression in the adult mouse brain. *Nature* **445**, 168–176 (2007). 17151600 10.1038/nature05453

[45] McCormick, D. A., Nestvogel, D. B. & He, B. J. Neuromodulation of brain state and behavior. *Annu. Rev. Neurosci.* **43**, 391–415 (2020). 32250724 10.1146/annurev-neuro-100219-105424 PMC12237593

[46] Lee, S.-H. & Dan, Y. Neuromodulation of brain states. *Neuron* **76**, 209–222 (2012). 23040816 10.1016/j.neuron.2012.09.012 PMC3579548

[47] Ghosh, A. et al. Locus coeruleus activation patterns differentially modulate odor discrimination learning and odor valence in rats. *Cereb. Cortex Commun.* **2**, tgab026 (2021). 34296171 10.1093/texcom/tgab026 PMC8152946

[48] Giorgi, F. S. et al. Locus coeruleus and neurovascular unit: from its role in physiology to its potential role in Alzheimer’s disease pathogenesis. *J. Neurosci. Res.* **98**, 2406–2434 (2020). 32875628 10.1002/jnr.24718

[49] Devor, A. et al. Coupling of the cortical hemodynamic response to cortical and thalamic neuronal activity. *Proc. Natl Acad. Sci. USA* **102**, 3822–3827 (2005). 15734797 10.1073/pnas.0407789102 PMC550644

[50] Moon, H. S. et al. Contribution of excitatory and inhibitory neuronal activity to BOLD fMRI. *Cereb. Cortex* **31**, 4053–4067 (2021). 33895810 10.1093/cercor/bhab068 PMC8328221

[51] Commons, K. G. Neuronal pathways linking substance P to drug addiction and stress. *Brain Res.* **1314**, 175–182 (2010). 19913520 10.1016/j.brainres.2009.11.014 PMC4028699

[52] Vazquez, A. L., Fukuda, M. & Kim, S.-G. Inhibitory neuron activity contributions to hemodynamic responses and metabolic load examined using an inhibitory optogenetic mouse model. *Cereb. Cortex* **28**, 4105–4119 (2018). 30215693 10.1093/cercor/bhy225 PMC6188559

[53] Katz, B. M., Walton, L. R., Houston, K. M., Cerri, D. H. & Shih, Y.-Y. I. Putative neurochemical and cell type contributions to hemodynamic activity in the rodent caudate putamen. *J. Cereb. Blood Flow Metab.* **43**, 481–498 (2023). 36448509 10.1177/0271678X221142533 PMC10063835

[54] Takata, N. et al. Optogenetic astrocyte activation evokes BOLD fMRI response with oxygen consumption without neuronal activity modulation. *Glia* **66**, 2013–2023 (2018). 29845643 10.1002/glia.23454

[55] Nizar, K. et al. In vivo stimulus-induced vasodilation occurs without IP3 receptor activation and may precede astrocytic calcium increase. *J. Neurosci.* **33**, 8411–8422 (2013). 23658179 10.1523/JNEUROSCI.3285-12.2013 PMC3712855

[56] Bekar, L. K., He, W. & Nedergaard, M. Locus coeruleus α-adrenergic-mediated activation of cortical astrocytes in vivo. *Cereb. Cortex* **18**, 2789–2795 (2008). 18372288 10.1093/cercor/bhn040 PMC2583159

[57] Vezoli, J. et al. Cortical hierarchy, dual counterstream architecture and the importance of top-down generative networks. *Neuroimage* **225**, 117479 (2021). 33099005 10.1016/j.neuroimage.2020.117479 PMC8244994

[58] Totah, N. K., Neves, R. M., Panzeri, S., Logothetis, N. K. & Eschenko, O. The locus coeruleus is a complex and differentiated neuromodulatory system. *Neuron* **99**, 1055–1068 (2018). 30122373 10.1016/j.neuron.2018.07.037

[59] Vazey, E. M. & Aston-Jones, G. Designer receptor manipulations reveal a role of the locus coeruleus noradrenergic system in isoflurane general anesthesia. *Proc. Natl Acad. Sci. USA* **111**, 3859–3864 (2014). 24567395 10.1073/pnas.1310025111 PMC3956184

[60] McGinley, M. J. et al. Waking state: rapid variations modulate neural and behavioral responses. *Neuron* **87**, 1143–1161 (2015). 26402600 10.1016/j.neuron.2015.09.012 PMC4718218

[61] Schwarz, L. A. & Luo, L. Organization of the locus coeruleus-norepinephrine system. *Curr. Biol.* **25**, R1051–R1056 (2015). 26528750 10.1016/j.cub.2015.09.039

[62] Parlato, R., Otto, C., Begus, Y., Stotz, S. & Schütz, G. Specific ablation of the transcription factor CREB in sympathetic neurons surprisingly protects against developmentally regulated apoptosis. *Development* **134**, 1663–1670 (2007). 17376811 10.1242/dev.02838

[63] Privitera, M. et al. A complete pupillometry toolbox for real-time monitoring of locus coeruleus activity in rodents. *Nat. Protoc.* **15**, 2301–2320 (2020). 32632319 10.1038/s41596-020-0324-6

[64] Yang, L., Lee, K., Villagracia, J. & Masmanidis, S. C. Open source silicon microprobes for high throughput neural recording. *J. Neural Eng.* **17**, 016036 (2020). 31731284 10.1088/1741-2552/ab581a PMC7227378

[65] Grimm, C., Wenderoth, N. & Zerbi, V. An optimized protocol for assessing changes in mouse whole-brain activity using opto-fMRI. *STAR Protoc.* **3**, 101761 (2022). 36240060 10.1016/j.xpro.2022.101761 PMC9568887

[66] Mathis, A. et al. DeepLabCut: markerless pose estimation of user-defined body parts with deep learning. *Nat. Neurosci.* **21**, 1281–1289 (2018). 30127430 10.1038/s41593-018-0209-y

[67] Nath, T. et al. Using DeepLabCut for 3D markerless pose estimation across species and behaviors. *Nat. Protoc.* **14**, 2152–2176 (2019). 31227823 10.1038/s41596-019-0176-0

[68] Pachitariu, M., Steinmetz, N., Kadir, S., Carandini, M. & Harris, K. D. Kilosort: realtime spike-sorting for extracellular electrophysiology with hundreds of channels. Preprint at *bioXiv*10.1101/061481 (2016).

[69] Avants, B. B. et al. A reproducible evaluation of ANTs similarity metric performance in brain image registration. *Neuroimage* **54**, 2033–2044 (2011). 20851191 10.1016/j.neuroimage.2010.09.025 PMC3065962

[70] Eklund, A., Nichols, T. E. & Knutsson, H. Cluster failure: why fMRI inferences for spatial extent have inflated false-positive rates. *Proc. Natl Acad. Sci. USA* **113**, 7900–7905 (2016). 27357684 10.1073/pnas.1602413113 PMC4948312

[71] Woo, C.-W., Krishnan, A. & Wager, T. D. Cluster-extent based thresholding in fMRI analyses: pitfalls and recommendations. *Neuroimage* **91**, 412–419 (2014). 24412399 10.1016/j.neuroimage.2013.12.058 PMC4214144

[72] Rubinov, M. & Sporns, O. Complex network measures of brain connectivity: uses and interpretations. *Neuroimage* **52**, 1059–1069 (2010). 19819337 10.1016/j.neuroimage.2009.10.003

[73] Grimm, C. et al. Optogenetic activation of striatal D1R and D2R cells differentially engages downstream connected areas beyond the basal ganglia. *Cell Rep.* **37**, 110161 (2021). 34965430 10.1016/j.celrep.2021.110161

[74] Grimm, C. et al. Locus coeruleus firing patterns selectively modulate brain activity and dynamics. *Zenodo* https://zenodo.org/records/7064020#.Yyw2ri8Rpjl (2022).

