# Supplementary material for: Tonic and burst-like locus coeruleus stimulation distinctly shift network activity across the cortical hierarchy
Source: Nat Neurosci. 2024 Sep 16;27(11):2167–77. doi: 10.1038/s41593-024-01755-8 (PMC11537968; doi:10.1038/s41593-024-01755-8)
Supplement: Supplementary file 1 — Supplementary Table 1. [file 41593_2024_1755_MOESM1_ESM.pdf]

nature neuroscience

Supplementary information

https://doi.org/10.1038/s41593-024-01755-8

Tonic and burst-like locus coeruleus stimulation distinctly shift network activity across the cortical hierarchy

In the format provided by the authors and unedited

Supplementary Information

Supplementary Table 1 Correlation between LC group stimulation maps and adrenergic receptor density maps.

	Noradrenaline			Dopamine				Serotonin					Acetylcholine					Zerbi et al., 2019
	Adra1a	Adra2a	Adrb	Drd1	Drd2	Drd3	Drd4	Htr1	Htr2	Htr3	Htr4	Htr5	Chrm1	Chrm2	Chrm3	Chrm4	Chrm5	NMI
sham	-0.11	-0.16	-0.06	-0.05	-0.01	-0.09	-0.16	-0.07	0.13	-0.07	0.04	-0.07	-0.08	0.04	-0.14	-0.001	0.04	-0.12
3 Hz	0.33*	0.08	0.25	0.25	0.23	-0.009	0.24	0.34	-0.08	0.19	0.19	0.19	-0.05	0.004	0.04	0.002	0.02	0.17
15 Hz	0.30*	0.11	0.23	0.23	0.30*	0.006	0.17	0.27	-0.024	0.22	0.25	0.21	-0.0003	-0.05	0.07	0.06	0.06	0.18
5 Hz	0.34*	0.15	0.38*	0.38*	0.37*	0.07	0.20	0.47*	0.04	0.30*	0.24	0.26*	-0.02	0.004	0.04	0.03	0.03	0.33*

Correlation LC stimulation group activation maps (cluster corr.) and voxelwise density maps of alpha1-2 adrenergic receptors (Adra1a, Adra2a subtypes), beta adrenergic receptors (Adrb), dopamine receptors 1-4 (Drd1-4), serotonin receptors 1-5 (Htr1-5), and cholinergic receptors 1-5 (Chrm1-5) (Spearman's ρ; *p<0.001 adjusted, two-tailed, Bonferroni corrected) as well as with changes in overall connectivity strength upon chemogenetic LC activation as quantified by a node modulation index (NMI) in [23] (Spearman's ρ; *p<0.001 adjusted, two-tailed, Bonferroni corrected)). N(sham)=32; n(3 Hz)=15; n(5 Hz)=16; n(15 Hz)=18.
