# Supplementary material for: Tonic and burst-like locus coeruleus stimulation distinctly shift network activity across the cortical hierarchy
Source: Nat Neurosci. 2024 Sep 16;27(11):2167–77. doi: 10.1038/s41593-024-01755-8 (PMC11537968; doi:10.1038/s41593-024-01755-8)
Supplement: Supplementary file 2 — Reporting Summary [file 41593_2024_1755_MOESM2_ESM.pdf]

nature portfolio

Corresponding author(s): Valerio Zerbi

Last updated by author(s): Jun 21, 2024

Reporting Summary

Nature Portfolio wishes to improve the reproducibility of the work that we publish. This form provides structure for consistency and transparency in reporting. For further information on Nature Portfolio policies, see our Editorial Policies and the Editorial Policy Checklist.

Statistics

For all statistical analyses, confirm that the following items are present in the figure legend, table legend, main text, or Methods section.

n/a	Confirmed	
☐	☒	The exact sample size (*n*) for each experimental group/condition, given as a discrete number and unit of measurement
☐	☒	A statement on whether measurements were taken from distinct samples or whether the same sample was measured repeatedly
☐	☒	The statistical test(s) used AND whether they are one- or two-sided<br>*Only common tests should be described solely by name; describe more complex techniques in the Methods section.*
☒	☐	A description of all covariates tested
☐	☒	A description of any assumptions or corrections, such as tests of normality and adjustment for multiple comparisons
☐	☒	A full description of the statistical parameters including central tendency (e.g. means) or other basic estimates (e.g. regression coefficient) AND variation (e.g. standard deviation) or associated estimates of uncertainty (e.g. confidence intervals)
☐	☒	For null hypothesis testing, the test statistic (e.g. *F*, *t*, *r*) with confidence intervals, effect sizes, degrees of freedom and *P* value noted<br>*Give P values as exact values whenever suitable.*
☒	☐	For Bayesian analysis, information on the choice of priors and Markov chain Monte Carlo settings
☒	☐	For hierarchical and complex designs, identification of the appropriate level for tests and full reporting of outcomes
☐	☒	Estimates of effect sizes (e.g. Cohen's *d*, Pearson's *r*), indicating how they were calculated

Our web collection on statistics for biologists contains articles on many of the points above.

Software and code

Policy information about availability of computer code

Data collection	ParaVision (V 6.1); DeepLabCut (V 2.1.7.1); Doric Neuroscience Studio (V 6.1); Bonsai (V 2.6.2);<br>All MRI data used in this paper are openly available at this address: https://zenodo.org/record/7064020#.Yyw2ri8Rpjl
Data analysis	Pupillometry App (https://ethz-ins.shinyapps.io/pupillometry/); MATLAB (V 2017b, 2021a & b); Kilosort 3; FSL (V 5.92); ANTs (V 2.1.0)

For manuscripts utilizing custom algorithms or software that are central to the research but not yet described in published literature, software must be made available to editors and reviewers. We strongly encourage code deposition in a community repository (e.g. GitHub). See the Nature Portfolio guidelines for submitting code & software for further information.

Data

Policy information about availability of data

All manuscripts must include a data availability statement. This statement should provide the following information, where applicable:

- Accession codes, unique identifiers, or web links for publicly available datasets
- A description of any restrictions on data availability
- For clinical datasets or third party data, please ensure that the statement adheres to our policy

Data supporting the findings of this study are available within the paper and its Supplementary Information files. Raw MRI data are openly available with this paper at the address:
https://zenodo.org/record/7064020#.Yyw2ri8Rpjl . Anatomical regions of the mouse brain were chosen based on the Mouse Brain Allen Common Coordinate

Framework v3
(CCFv3; https://connectivity.brain-map.org/static/referencedata#:~:text=The%20Allen%20Mouse%20Brain%20Common,using%20serial%20two%2Dphoton%20tomography.)

Research involving human participants, their data, or biological material

Policy information about studies with human participants or human data. See also policy information about sex, gender (identity/presentation), and sexual orientation and race, ethnicity and racism.

Reporting on sex and gender	N/A
Reporting on race, ethnicity, or other socially relevant groupings	N/A
Population characteristics	N/A
Recruitment	N/A
Ethics oversight	N/A

Note that full information on the approval of the study protocol must also be provided in the manuscript.

Field-specific reporting

Please select the one below that is the best fit for your research. If you are not sure, read the appropriate sections before making your selection.

☒ Life sciences ☐ Behavioural & social sciences ☐ Ecological, evolutionary & environmental sciences

For a reference copy of the document with all sections, see nature.com/documents/nr-reporting-summary-flat.pdf

Life sciences study design

All studies must disclose on these points even when the disclosure is negative.

Sample size	The estimation of the number of animals is based on our previous experience and prior work in rodent fMRI (1,2,3). The studies were conducted with n> With a group size of n=15 an effect size of 20% can be detected, assuming an inter-individual variation (SD) of 30%, a p<=0.05 and a power (1-beta) of 0.8.<br><br>1. Zerbi, V. et al. Rapid Reconfiguration of the Functional Connectome after Chemogenetic Locus Coeruleus Activation. Neuron 103, 702- 718.e5 (2019).<br>2. Grimm, C., Wenderoth, N. & Zerbi, V. An optimized protocol for assessing changes in mouse whole-brain activity using opto-fMRI. STAR Protoc. 3, 101761 (2022).<br>3. Grimm, C. et al. Optogenetic activation of striatal D1R and D2R cells differentially engages downstream connected areas beyond the basal ganglia. Cell Rep. 37, 110161 (2021).
Data exclusions	Data of animals were excluded if no pupil response to optogenetic stimulations was present. Furthermore, fMRI datasets were excluded in case of fMRI coil-related artefacts.
Replication	To ensure reproducibility of experimental findings, experimental settings including targeting areas, working material, anesthesia levels, time under anesthesia, and recording areas were kept the same for each eindividual xperimental modality. Furthermore, animal age and weight were matched across experimental modalities. This way, all attempts at replication were successful.
Randomization	Scan sessions with different optogenetic stimulation were randomly assigned to all mice. For pupillometry, photometry, and electrophysiological experiments, all animals were allocated randomly to each optogenetic stimulation protocol.
Blinding	The experimentor was not blind to the type of optogenetic stimlation used, as the correct stimulation sequence had to be programmed before fMRI, pupillometry, and photometry recordings.

Reporting for specific materials, systems and methods

We require information from authors about some types of materials, experimental systems and methods used in many studies. Here, indicate whether each material, system or method listed is relevant to your study. If you are not sure if a list item applies to your research, read the appropriate section before selecting a response.

Materials & experimental systems

n/a	Involved in the study
☐	☒ Antibodies
☒	☐ Eukaryotic cell lines
☒	☐ Palaeontology and archaeology
☐	☒ Animals and other organisms
☒	☐ Clinical data
☒	☐ Dual use research of concern
☒	☐ Plants

Methods

n/a	Involved in the study
☒	☐ ChIP-seq
☒	☐ Flow cytometry
☐	☒ MRI-based neuroimaging

Antibodies

Antibodies used

Mouse anti-TH (22941, Immunostar), chicken anti-GFP (ab13970, Abcam), and rabbit anti-cFOS (226 003, Synaptic Systems), donkey anti-mouse Alexa 647
(A-31571, Thermo Fisher Scientific), goat anti-chicken Alexa Fluor 488 (A-11039, Thermo Fischer Scientific), and goat anti-rabbit Alexa 546 (A11035, Thermo Fisher Scientific)

Validation

Immunohistological stainings using above mentioned antibodies were confirmed using a confocal laser-scanning microscope (CLSM 880, Carl Zeiss AG, Germany).

Animals and other research organisms

Policy information about studies involving animals; ARRIVE guidelines recommended for reporting animal research, and Sex and Gender in Research

Laboratory animals

Mice, heterozygous C57BL/6-Tg(Dbh-iCre)1Gsc (DBH-iCre), age: 2-3 months; mice, NET-cre::Ai148 (GCaMP6f), age: 8-9 weeks

Wild animals

No wild animals were used in this study.

Reporting on sex

We used heterozygous male (n=22) and female (n=7) DBH-iCre mice. In a separate cohort of DBH-iCre mice, immunohistochemical validation of LC targeting was verified (n(male)=11).
We used male NET-cre::Ai148 (GCaMP6f; n=3) mice. We report sex in our data.

Field-collected samples

No field-collected samples were used in this study.

Ethics oversight

All animal procedures were conducted in accordance with the Swiss federal guidelines for the use of animals in research and approved by the Cantonal Veterinary Office of Zurich.

Note that full information on the approval of the study protocol must also be provided in the manuscript.

Plants

Seed stocks

N/A

Novel plant genotypes

N/A

Authentication

N/A

Magnetic resonance imaging

Experimental design

Design type

Block design; optogenetics-fMRI

Design specifications

In each scan, trains of 473 nm laser pulses were delivered for 30 sec above the targeted site, followed by 30 sec of no laser light delivery for nine times

Behavioral performance measures: N/A

Acquisition

Imaging type(s): Functional imaging

Field strength: 7T

Sequence & imaging parameters: For anatomical assessment, a T1-weighted image was acquired via a FLASH sequence with an in-plane resolution of 0.05 x 0.02 mm2, an echo time (TE) of 3.51 ms and a repetition time (TR) of 522 ms. For functional scans, a standard gradient-echo echo-planar imaging sequence (GE-EPI, repetition time TR = 1 s, echo time TE = 15ms, in-plane resolution RES = 0.22 × 0.2mm2, number of slice NS = 20, slice thickness ST = 0.4 mm, slice gap = 0.1mm) was applied to acquire 1440 volumes in 24 min.

Area of acquisition: Whole-brain scans

Diffusion MRI: ☐ Used ☒ Not used

Preprocessing

Preprocessing software: Preprocessing and functional data analysis was carried out using FSL FEAT.

Normalization: To account for potential alignment artefacts due to the implanted optical fiber, two study-specific templates based on all mean-EPIs and T1-weighted anatomical images were created using ANTs (version 2.1.0; http://stnava.github.io/ANTs/). Registration was carried out first to the study-specific EPI template and then to the T1-weighted template using FLIRT and FNIRT.

Normalization template: We used a study-specific standard space template. For visualization purposes, we used the Allen Common Coordinate Framework Atlas (CCv3).

Noise and artifact removal: N/A

Volume censoring: N/A

Statistical modeling & inference

Model type and settings: Mass univariate; FSL FEAT; fixed effects

Effect(s) tested: Stimulation intensities (3Hz vs 5Hz vs Sham), Stimulation type (3Hz vs 15Hz vs Sham)

Specify type of analysis: ☐ Whole brain ☐ ROI-based ☒ Both

Anatomical location(s): Manually defined and defined based on Allen Brain Atlas parcellation.

Statistic type for inference (See Eklund et al. 2016): Cluster-wise, Z-statistic images were thresholded using clusters determined by z>3.1.

Correction: Familywise error–corrected cluster significance threshold of p<0.05 was applied to the suprathreshold clusters

Models & analysis

n/a	Involved in the study	
☒	☐	Functional and/or effective connectivity
☒	☐	Graph analysis
☒	☐	Multivariate modeling or predictive analysis
